# GnRH-driven FTO-mediated RNA m^6^A modification promotes gonadotropin synthesis and secretion

**DOI:** 10.1186/s12915-024-01905-1

**Published:** 2024-05-03

**Authors:** Hao-Qi Wang, Yi-Ran Ma, Yu-Xin Zhang, Fan-Hao Wei, Yi Zheng, Zhong-Hao Ji, Hai-Xiang Guo, Tian Wang, Jia-Bao Zhang, Bao Yuan

**Affiliations:** Department of Laboratory Animals, College of Animal Sciences, Jilin University, Changchun, Jilin 130062 P.R. China

**Keywords:** Pituitary, Gonadotropin, m^6^A modification, FTO, Reproduction

## Abstract

**Background:**

Gonadotropin precisely controls mammalian reproductive activities. Systematic analysis of the mechanisms by which epigenetic modifications regulate the synthesis and secretion of gonadotropin can be useful for more precise regulation of the animal reproductive process. Previous studies have identified many differential m^6^A modifications in the GnRH-treated adenohypophysis. However, the molecular mechanism by which m^6^A modification regulates gonadotropin synthesis and secretion remains unclear.

**Results:**

Herein, it was found that GnRH can promote gonadotropin synthesis and secretion by promoting the expression of FTO. Highly expressed FTO binds to *Foxp2* mRNA in the nucleus, exerting a demethylation function and reducing m^6^A modification. After *Foxp2* mRNA exits the nucleus, the lack of m^6^A modification prevents YTHDF3 from binding to it, resulting in increased stability and upregulation of *Foxp2* mRNA expression, which activates the cAMP/PKA signaling pathway to promote gonadotropin synthesis and secretion.

**Conclusions:**

Overall, the study reveals the molecular mechanism of GnRH regulating the gonadotropin synthesis and secretion through FTO-mediated m^6^A modification. The results of this study allow systematic interpretation of the regulatory mechanism of gonadotropin synthesis and secretion in the pituitary at the epigenetic level and provide a theoretical basis for the application of reproductive hormones in the regulation of animal artificial reproduction.

**Supplementary Information:**

The online version contains supplementary material available at 10.1186/s12915-024-01905-1.

## Background

The hypothalamic-pituitary–gonadal axis precisely controls reproductive activity in mammals [1]. Gonadotropin-releasing hormone (GnRH), released in a pulsed manner by the hypothalamus, regulates sexual development, gametogenesis, and other reproductive activities by regulating the synthesis and secretion of gonadotropins. Gonadotropins are synthesized and secreted by gonadotrophic cells (a type of basophil) in the adenohypophysis [2]. It is transported through the peripheral blood circulation to the appropriate target organs and performs its biological functions. Follicle-stimulating hormone (FSH) acts synergistically with luteinizing hormone (LH) under physiological conditions. They act through specific G protein-coupled receptors (GPCRs), namely, follicle-stimulating hormone receptor (FSHR) and luteinizing hormone/choriogonadotropin receptor (LHCGR), which activate different signaling cascades to regulate physiological activities such as steroid hormone production, cell metabolism, and growth [3]. In addition, FSHR has also been shown to be expressed and exert potential biological functions in extragonadal organs and tumor tissues [4]. Currently, gonadotropins and their analogs are widely used in the regulation of artificial reproduction in animals and assisted reproduction in humans [5]. However, the effects of their application in practical production remain unstable, and the relevant theoretical and technical “bottlenecks” remain unresolved due to the limited knowledge of the mechanism of GnRH regulation of gonadotropin synthesis and secretion. It is necessary to systematically analyze the mechanism of gonadotropin synthesis and secretion, which will help to regulate animal reproduction more accurately, complete human-assisted reproduction more efficiently, and improve the artificial regulation of animal reproduction in a more systematic manner.

It is a complex mechanism for the regulation of gonadotropin synthesis and secretion. A key step in this process is the transcriptional regulation and posttranscriptional modification of *Fshb* and *Lhb*. GnRH has been shown to act on the gonadotropin-releasing hormone receptor (GnRHR) of gonadotrophic cells [6]. Activated GnRHR promotes the inward flow of extracellular Ca^2+^ and activates G proteins. This further activates cAMP/PKA, PKC/MAPK, Ca^2+^/CaMKII, and other downstream classic signaling pathways to regulate gonadotropin biosynthesis and secretion in different modes [7–10]. The AP-1 [11–13], CREB [14, 15], FOXL2 [16, 17], and Nur77 [18] transcription factors are also able to respond to signals transduced by the above pathways and are participate in regulating the transcription of *Fshb* and *Lhb*. In recent years, with continued epigenetic research, the molecular mechanisms regulating gonadotropins are no longer limited to intergenic regulation and protein activation and interactions. The authors and their team have demonstrated in previous studies that miR-7a-5p [19], lncRNA-m433s1 [20], lncRNA-m18as1 [21], circAkap17b [22], and other non-coding RNAs can participate in the regulation of FSH synthesis and secretion. The above findings suggest that there are many unknown posttranscriptional regulatory mechanisms in the process of gonadotropin synthesis and secretion, which need to be deeply explored.

N6-methyladenosine (m^6^A) modification is becoming a hot topic in the field of epitranscriptomics with the development of epigenetic research [23–26]. The gradual discovery of methyltransferases (writers), demethylases (erasers), and methylation reader proteins (readers), which dynamically regulate m^6^A modifications, has led to a better understanding of the process and biological functions of m^6^A modifications [27]. It has been shown that mRNA splicing, translocation, degradation, and translation are all affected by m^6^A modifications, confirming that such posttranscriptional modifications play a potential role in a variety of physiological activities.

There is mounting evidence that the normal collaboration of m^6^A-related enzymes and the dynamic balance of m^6^A modifications are necessary to maintain normal reproductive activity during the complex process of reproductive phylogeny and gametogenesis. In males, deletion of the methyltransferases METTL3 and METTL14 [28], and the demethylase ALKBH5 [29] can lead to varying degrees of disorder in spermatogenesis. Abnormalities in m^6^A modification can lead to abnormal spermatogenesis and infertility [30, 31]. In females, deletion of METTL3 interferes with the normal expression of key genes for gonadotropin signaling and sex hormone synthesis, which in turn affects oocyte maturation quality and fertility [32]. All of the above studies suggest that m^6^A modification may play a potential biological role in the synthesis and secretion of reproductive hormones. However, there are few studies on m^6^A modification in reproductive hormones. There is no detailed report on the mechanism of m^6^A modification and related enzymes regulating gonadotropin synthesis and secretion. The functions and regulatory networks of m^6^A modification in pituitary hormone secretion are also still unclear. Therefore, it is important to investigate the molecular mechanism of m^6^A modification in the regulation of gonadotropin synthesis and secretion to gain a better understanding of molecular endocrinology and to achieve more accurate artificial regulation of animal reproduction.

FTO, as the first identified demethylase, is able to perform its demethylation function in the circulatory system to alleviate the degradation of cardiac contraction-related transcripts such as *Nppa* and *Myh7*, thereby maintaining cardiac homeostasis [33]. In the central nervous system, FTO deficiency directly affects the proliferation of neuronal stem cells and neuronal differentiation, which in turn leads to impaired learning ability and memory [34]. Research on the vital activities of FTO-mediated m^6^A modifications provides new and promising ideas for investigating the functions of m^6^A modifications in depth. In a previous study, the authors found that many differences in m^6^A modification were observed in the adenohypophysis after GnRH treatment and that the expression of *Fto* mRNA was reduced [35]. However, m^6^A modifications on *Fshb* and *Lhb* mRNA did not appear significantly different under GnRH stimulation. This suggests that FTO does not participate in the regulation of gonadotropin synthesis and secretion by directly regulating m^6^A modifications on *Fshb* and *Lhb* mRNA, but may exist as a mediator. Therefore, in this study, adenohypophysis m^6^A modification was examined to analyze the regulatory role and molecular mechanism of FTO-mediated m^6^A modifications on gonadotropin synthesis and secretion. The results of this study are expected to provide a theoretical basis for the regulation of artificial reproduction in animals and the application of reproductive hormones in human-assisted reproduction.

## Results

### GnRH downregulates the level of m^6^A modification and upregulates the expression of FTO in gonadotropin cells

**Fig. 1:**
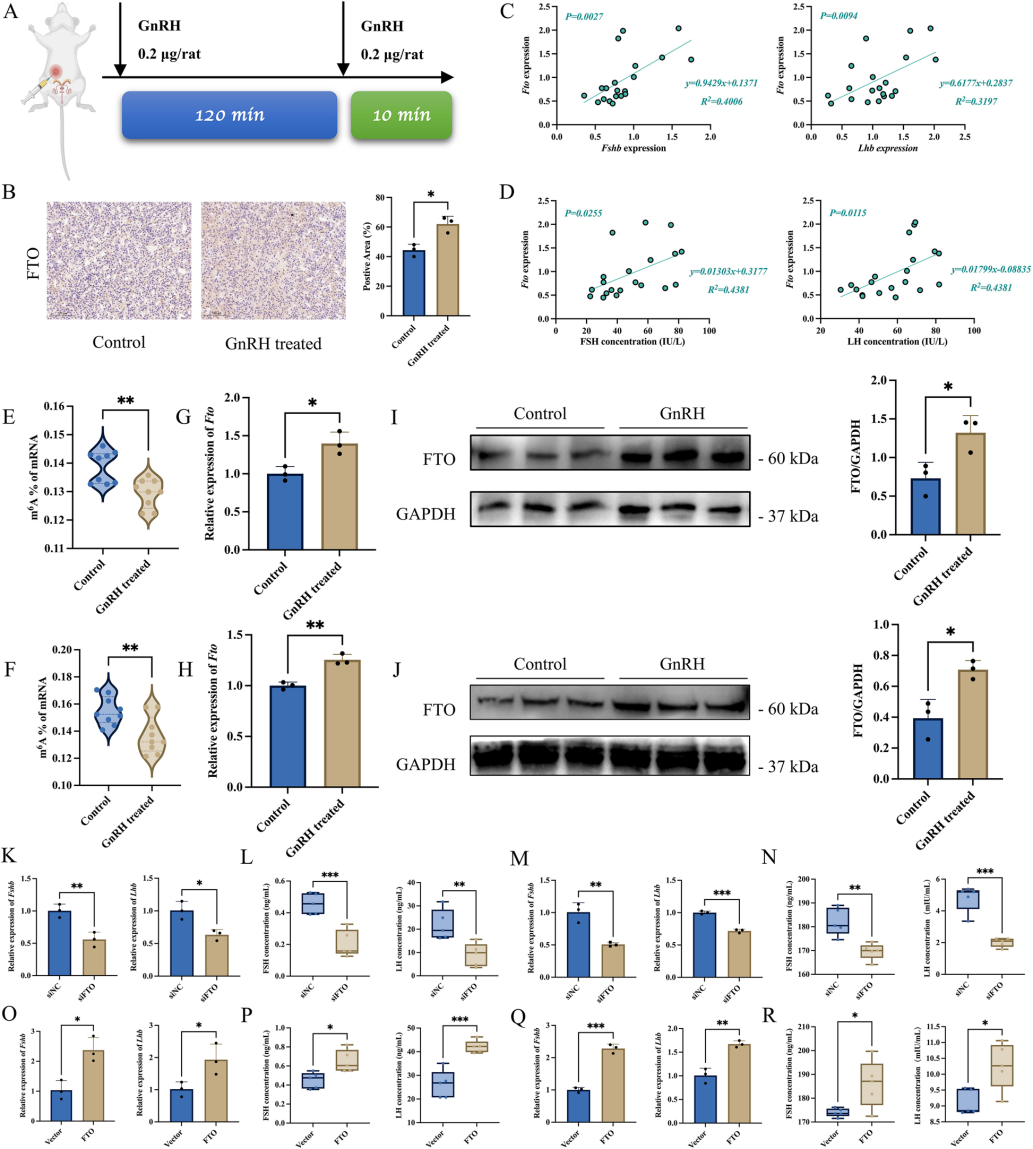
GnRH promotes gonadotropin synthesis and secretion by upregulating FTO expression. **A** Pattern diagram of the twice GnRH treatment procedures. **B** Immunohistochemical detection of FTO protein levels in rat adenohypophysis after GnRH treatment. Positive signal regions of FTO protein were analyzed using ImageJ (*n* = 3). **C** Correlation analysis of *Fto* mRNA expression and *Fshb* (left)*/Lhb* (right) mRNA expression in rats (*n* = 20). **D** Correlation analysis of *Fto* mRNA expression and FSH (left)/LH (right) secretion level in rats (*n* = 20). **E**, **F** Effect of GnRH on the level of total RNA m.^6^A modification in LβT2 cells (**E**, *n* = 9) and primary rat adenohypophysis cells (**F**, *n* = 9). **G**, **H** RT-qPCR analysis *Fto* mRNA expression after GnRH treatment in LβT2 cells (**G**, *n* = 3) and primary rat adenohypophysis cells (**H**, *n* = 3). **I**, **J** WB analysis FTO protein expression after GnRH treatment in LβT2 cells (**I**, *n* = 3) and primary rat adenohypophysis cells (**J**, *n* = 3). **K**-**N** RT-qPCR analysis *Fshb* and *Lhb* mRNA expression transfected with siNC (siNC group) or FTO siRNA (siFTO group) in LβT2 cells (**K**, *n* = 3) and primary rat adenohypophysis cells (**M**, *n* = 3), respectively. ELISA analysis secretion levels of FSH and LH transfected with siNC (siNC group) or FTO siRNA (siFTO group) in LβT2 cells (**L**, *n* = 5) and primary rat adenohypophysis cells (**N**, *n* = 5), respectively. **O**-**R** RT-qPCR analysis *Fshb* and *Lhb* mRNA expression transfected with vector (Vector group) or FTO overexpression plasmid (FTO group) in LβT2 cells (**O**, *n* = 3) and primary rat adenohypophysis cells (**Q**, *n* = 3), respectively. ELISA analysis secretion levels of FSH and LH transfected with vector (Vector group) or FTO overexpression plasmid (FTO group) in LβT2 cells (**P**, *n* = 5) and primary rat adenohypophysis cells (**R**, *n* = 5), respectively. *, *P* < 0.05; **, *P* < 0.01; ***, *P* < 0.001

In vivo, two treatments of 0.2 μg of GnRH were administered to 8-week-old male SD rats in conjunction with the basis of previous studies to simulate the pulsatile release pattern of GnRH (Fig. 1A). The results showed that GnRH treatment did not affect the overall number, cell arrangement, or other morphological structures of the adenohypophysis cells (Additional file 1: Fig. S1B). However, GnRH significantly promoted the expression of *Fshb* mRNA and *Lhb* mRNA in the adenohypophysis (Additional file 1: Fig. S1C), and the secretion of FSH and LH in rats also appeared to be increased to different degrees under GnRH stimulation (Additional file 1: Fig. S1D). In addition, the expression levels of the FSHB protein in the adenohypophysis tissue were significantly increased after GnRH treatment (Additional file 1: Fig. S1E). The above results indicate that the GnRH treatment procedure used in the study can promote gonadotropin synthesis and secretion in rats. Since the authors' previous study had found that the expression of *Fto* mRNA was significantly elevated in the GnRH-treated rat adenohypophysis [35], further immunohistochemistry revealed that GnRH was able to significantly promote the expression of FTO protein in the adenohypophysis (Fig. 1B). In addition, the expression levels of *Fto* mRNA in the rat adenohypophysis were positively correlated with the expression of *Fshb* and *Lhb* mRNA (Fig. 1C). The expression levels of *Fto* mRNA in the adenohypophysis were also positively correlated with the secretion levels of FSH and LH in rats (Fig. 1D).

In vitro, GnRH treatments were performed on LβT2 cells and primary rat adenohypophysis cells. The results showed that GnRH significantly promoted the expression of *Fshb*, *Lhb*, and *Cga* mRNA (Additional file 1: Fig. S2A, B). FSH and LH secretion levels were also significantly increased (Additional file 1: Fig. S2C, D). Further assays revealed that GnRH significantly decreased the m^6^A modification level of total RNA in LβT2 cells (Fig. 1E) and primary rat adenohypophysis cells (Fig. 1F). The RT-qPCR results showed that GnRH significantly upregulated the expression level of *Fto* mRNA (Fig. 1G, H). Western blotting (WB) and immunofluorescence (IF) results showed that GnRH could significantly promote the expression of FTO protein (Fig. 1I, J, and Additional file 1: Fig. S2E). These data confirmed that GnRH could downregulate the level of m^6^A modification and upregulate the expression of FTO in gonadotropin cells.

### FTO promotes gonadotropin synthesis and secretion

To explore the potential function of FTO in the adenohypophysis and its regulation of gonadotropin synthesis and secretion, the siRNAs or overexpression plasmids of FTO were transfected into LβT2 cells and primary rat adenohypophysis cells, respectively. RT-qPCR and ELISA results showed that the knockdown of FTO significantly reduced *Fshb* and *Lhb* mRNA expression (Fig. 1K, M), and the secretion of FSH and LH was also significantly reduced (Fig. 1L, N). In contrast, overexpression of FTO significantly promoted the expression of *Fshb* and *Lhb* mRNA (Fig. 1O, Q), and the secretion of FSH and LH was also significantly increased (Fig. 1P, R).

### GnRH upregulates FOXP2 expression in gonadotrophic cells

To further clarify the molecular mechanism by which FTO regulates gonadotropin synthesis and secretion and to explore the key factors that are demethylated by FTO, we mined and analyzed omics data previously obtained by our group. The results of the pituitary single-cell transcriptome analysis showed that the *Foxp2* gene was specifically enriched in gonadotropin cells (Additional file 1: Fig. S3A). Further digging deeper into the m^6^A-seq results carried out in the previous period revealed that the *Foxp2* gene was highly expressed in GnRH-treated adenohypophysis tissues, and the level of m^6^A modification in the 3' UTR of its mRNA was significantly reduced in GnRH-treated adenohypophysis tissues (Additional file 1: Fig. S3B).

**Fig. 2:**
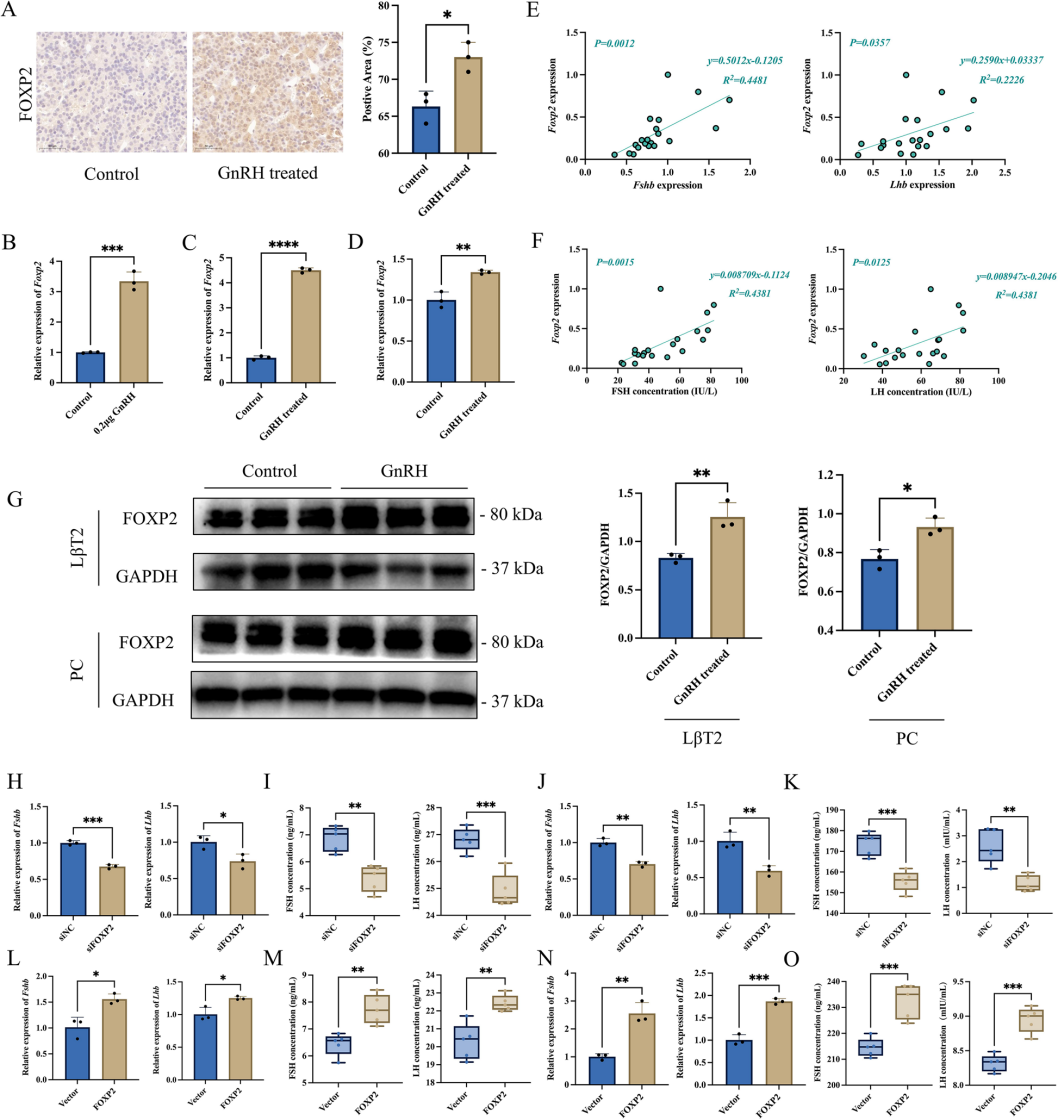
Highly expressed FOXP2 after stimulation by GnRH promotes gonadotropin synthesis and secretion. **A** Immunohistochemical detection of FOXP2 protein levels in rat adenohypophysis after GnRH treatment. Positive signal regions of FOXP2 protein were analyzed using ImageJ (*n* = 3). **B**-**D** RT-qPCR analysis *Foxp2* mRNA expression after GnRH treatment in rat adenohypophysis tissues (**B**, *n* = 3), primary rat adenohypophysis cells (**C**, *n* = 3) and LβT2 cells (**D**, *n* = 3). **E** Correlation analysis of *Foxp2* mRNA expression and *Fshb* (left)*/Lhb* (right) mRNA expression in rats (*n* = 20). **F** Correlation analysis of *Foxp2* mRNA expression and FSH (left)/LH (right) secretion level in rats (*n* = 20). **G** WB analysis FOXP2 protein expression after GnRH treatment in LβT2 cells and primary rat adenohypophysis cells (*n* = 3). **H**–**K** RT-qPCR analysis *Fshb* and *Lhb* mRNA expression transfected with siNC (siNC group) or FOXP2 siRNA (siFOXP2 group) in LβT2 cells (**H**, *n* = 3) and primary rat adenohypophysis cells (**J**, *n* = 3), respectively. ELISA analysis secretion levels of FSH and LH transfected with siNC (siNC group) or FOXP2 siRNA (siFOXP2 group) in LβT2 cells (I, n = 5) and primary rat adenohypophysis cells (**K**, *n* = 5), respectively. **L**-**O** RT-qPCR analysis *Fshb* and *Lhb* mRNA expression transfected with vector or FOXP2 overexpression plasmid in LβT2 cells (**L**, *n* = 3) and primary rat adenohypophysis cells (**N**, *n* = 3), respectively. ELISA analysis secretion levels of FSH and LH transfected with vector (Vector group) and FOXP2 overexpression plasmid (FOXP2 group) in LβT2 cells (**M**, *n* = 5) and primary rat adenohypophysis cells (**O**, *n* = 5), respectively. *, *P* < 0.05; **, *P* < 0.01; ***, *P* < 0.001; ****, *P* < 0.0001

The regulatory effects of GnRH on FOXP2 expression were further verified in vivo and in vitro by RT-qPCR, WB, and immunohistochemistry. The RT-qPCR results showed that GnRH treatment significantly promoted *Foxp2* mRNA expression in rat adenohypophysis tissues (Fig. 2B), primary rat adenohypophysis cells (Fig. 2C) and LβT2 cells (Fig. 2D). The results of WB and immunohistochemistry showed that GnRH treatment could significantly promote the expression of FOXP2 protein in rat adenohypophysis tissues (Fig. 2A), LβT2 cells (Fig. 2G), and primary rat adenohypophysis cells (Fig. 2G). In addition, the expression levels of *Foxp2* mRNA in the rat adenohypophysis were positively correlated with the expression of *Fshb* and *Lhb* mRNA (Fig. 2E). The expression levels of *Foxp2* mRNA in the adenohypophysis were also positively correlated with the secretion levels of FSH and LH in rats (Fig. 2F).

### FOXP2 regulates gonadotropin synthesis and secretion via the cAMP/PKA signaling pathway

To explore the potential function of FOXP2 in the adenohypophysis and its regulation of gonadotropin synthesis and secretion, the FOXP2 siRNAs or overexpression plasmids were transfected into LβT2 cells and primary rat adenohypophysis cells, respectively. RT-qPCR and ELISA results showed that the knockdown of FOXP2 significantly reduced *Fshb* and *Lhb* mRNA expression (Fig. 2H, J), and the secretion of FSH and LH was also significantly reduced (Fig. 2I, K). In contrast, overexpression of FOXP2 significantly promoted the mRNA expression of *Fshb* and *Lhb* (Fig. 2L, N), and the secretion of FSH and LH was also significantly increased (Fig. 2M, O).

To investigate the molecular mechanism through which FOXP2 regulates the synthesis and secretion of gonadotropins, the study first verified whether there was interplay between the FOXP2 protein and *Fshb* DNA by a yeast one-hybrid assay, in addition to the role of FOXP2 as a transcription factor. However, the results showed no interactions between the FOXP2 protein and *Fshb* DNA (Additional file 1: Fig. S4). In addition, flow cytometry results revealed that the apoptosis rate of LβT2 cells (Additional file 1: Fig. S5A, B) and primary rat adenohypophysis cells (Additional file 1: Fig. S5C, D) did not change significantly after knockdown or overexpression of FOXP2. This result suggests that FOXP2 does not exert its biological function by affecting the apoptosis of gonadotropin cells, and its molecular mechanism of regulating gonadotropin synthesis and secretion still needs to be further explored.

**Fig. 3:**
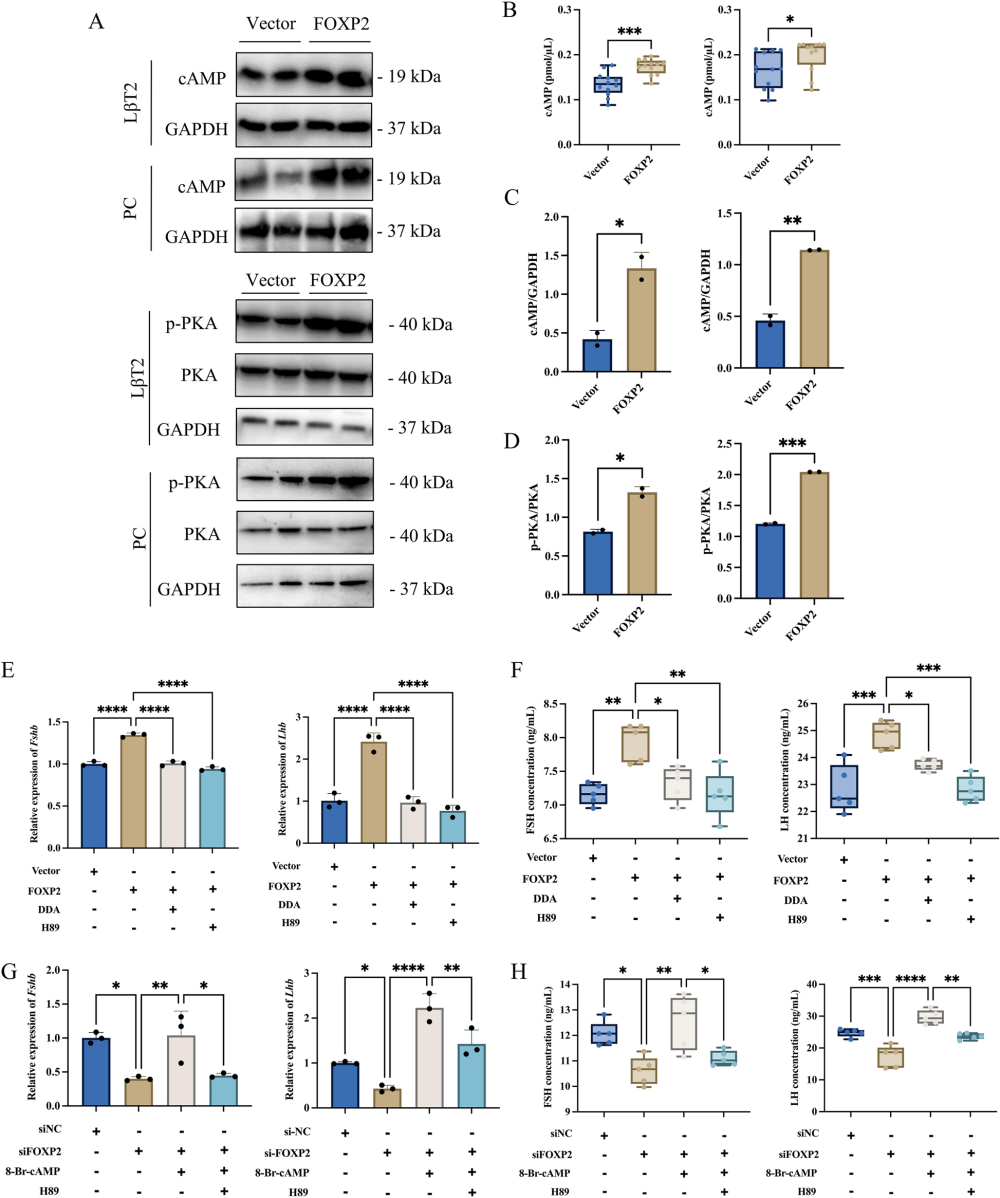
FOXP2 regulates gonadotropin synthesis and secretion via activating the cAMP/PKA signaling pathway. **A** WB analysis cAMP, PKA, and p-PKA transfected with vector (Vector group) or FOXP2 overexpression plasmid (FOXP2 group) in LβT2 cells and primary rat adenohypophysis cells. **B** Detection of intracellular cAMP levels transfected with vector (Vector group) or FOXP2 overexpression plasmid (FOXP2 group) in LβT2 cells (left) and primary rat adenohypophysis cells (right). **C** Statistical analysis of cAMP protein expression changes in LβT2 cells (left, *n* = 2) and primary rat adenohypophysis cells (right, *n* = 2). **D** Statistical analysis of p-PKA protein expression changes in LβT2 cells (left, n = 2) and primary rat adenohypophysis cells (right, *n* = 2). **E**, **F** FOXP2 overexpression plasmid or vector was transfected into LβT2 cells with or without the indicated compounds (DDA, 10 μM; H89, 10 μM). The expression of *Fshb* and *Lhb* mRNA was assayed by RT-qPCR (**E**, *n* = 3). The secretion of FSH and LH was assayed by ELISA (**F**, *n* = 5). **G**, **H** FOXP2 siRNA or siNC were transfected into LβT2 cells with or without the indicated compounds (8-Bromo-cAMP, 500 μM; H89, 10 μM). The expression of *Fshb* and *Lhb* mRNA was assayed by RT-qPCR (**G**, *n* = 3). The secretion of FSH and LH was assayed by ELISA (**H**, *n* = 5). *, *P* < 0.05; **, *P* < 0.01; ***, *P* < 0.001; ****, *P* < 0.0001

To investigate the specific molecular mechanism by which FOXP2 regulates gonadotropin synthesis and secretion, it was found that overexpression of FOXP2 significantly increased cAMP levels and phosphorylated PKA protein levels in LβT2 cells and primary rat adenohypophysis cells (Fig. 3A-D). The adenylate cyclase inhibitor DDA was cotreated with FOXP2 overexpression. RT-qPCR and ELISA results showed that inhibition of cAMP production significantly reduced the elevated levels of gonadotropin synthesis and secretion induced by overexpression of FOXP2 (Fig. 3E, F). Similarly, treatment with the PKA inhibitor H89 was performed simultaneously with FOXP2 overexpression. RT-qPCR and ELISA results showed that inhibition of PKA activity also significantly reduced the elevated gonadotropin synthesis and secretion levels induced by overexpression of FOXP2 (Fig. 3E, F). Furthermore, treatment with the PKA agonist 8-Bromo-cAMP was performed simultaneously with H89 treatments when FOXP2 was knocked down. RT-qPCR and ELISA results showed that 8-Bromo-cAMP was able to significantly revert to the reduced levels of gonadotropin synthesis and secretion induced by knocking down FOXP2 (Fig. 3G, H). The effect was attenuated by simultaneous inhibition of PKA activity (Fig. 3G, H). The same experiment was performed on primary rat adenohypophysis cells and same results were obtained (Additional file 1: Fig. S6). The above results indicated that FOXP2 was able to exert its promotional effect on gonadotropin synthesis and secretion by activating the cAMP/PKA signaling pathway.

### FTO promotes gonadotropin synthesis and secretion by promoting FOXP2 expression

**Fig. 4:**
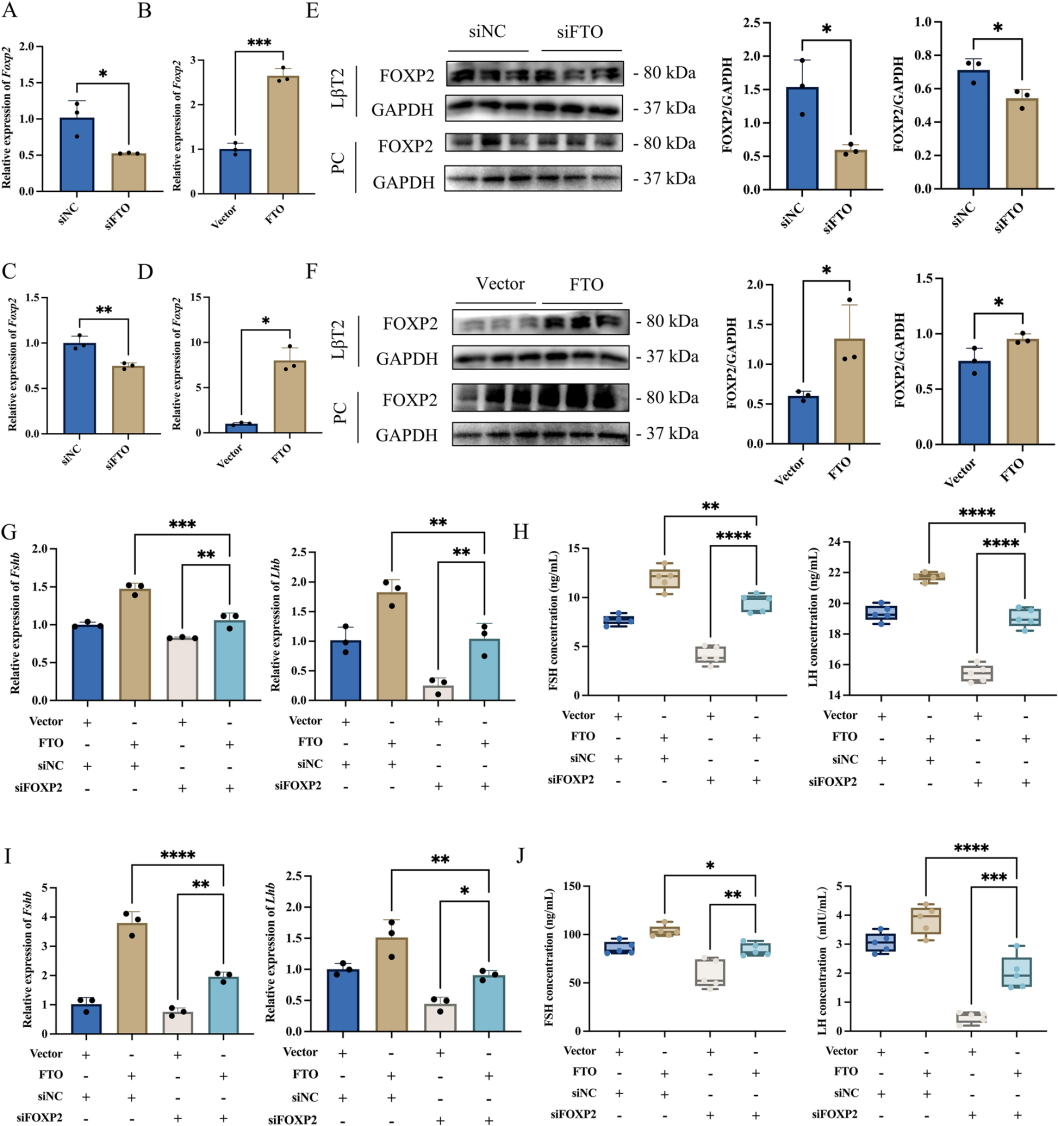
FTO promotes gonadotropin synthesis and secretion via promoting the expression of FOXP2. **A**-**D** RT-qPCR analysis *Foxp2* mRNA expression transfected with siNC (siNC group), FTO siRNA (siFTO group), vector (Vector group), or FTO overexpression plasmid (FTO group) in LβT2 cells (**A**, **B**, *n* = 3) and primary rat adenohypophysis cells (**C**, **D**, *n* = 3), respectively. **E** WB analysis FOXP2 transfected with siNC (siNC group) or FTO siRNA (siFTO group) in LβT2 cells and primary rat adenohypophysis cells and statistical analysis of FOXP2 protein expression changes in LβT2 cells (left, *n* = 3) and primary rat adenohypophysis cells (right, *n* = 3). **F** WB analysis FOXP2 transfected with vector (Vector group) or FTO overexpression plasmid (FTO group) and statistical analysis of FOXP2 protein expression changes in LβT2 cells (left, *n* = 3) and primary rat adenohypophysis cells (right, *n* = 3). **G**-**J** FTO overexpression plasmid was transfected with or without FOXP2 siRNA. The expression of *Fshb* and *Lhb* mRNA was assayed by RT-qPCR in LβT2 cells (**G**, *n* = 3) and primary rat adenohypophysis cells (**I**, *n* = 3). The secretion of FSH and LH was assayed by ELISA in LβT2 cells (**H**, *n* = 5) and primary rat adenohypophysis cells (**J**, *n* = 5). *, *P* < 0.05; **, *P* < 0.01; ***, *P* < 0.001; ****, *P* < 0.0001

To clarify whether FTO could exert its regulatory effect on gonadotropin synthesis and secretion by affecting the expression of FOXP2, FTO siRNA or overexpression plasmid were transfected into LβT2 cells and primary rat adenohypophysis cells, respectively. The RT-qPCR results showed that knockdown of FTO significantly decreased the expression of *Foxp2* mRNA (Fig. 4A, C), and overexpression of FTO significantly promoted the expression of *Foxp2* mRNA (Fig. 4B, D). The WB results also showed that knockdown of FTO significantly reduced the expression of FOXP2 protein (Fig. 4E), and overexpression of FTO significantly promoted the expression of FOXP2 protein (Fig. 4F). Rescue experiments were performed, in which FTO overexpression plasmid was transfected while co-transfected with FOXP2 siRNA on LβT2 cells and primary rat adenohypophysis cells. RT-qPCR and ELISA results showed that cotransfection was able to revert to the regulation of gonadotropin synthesis and secretion by FTO and FOXP2 (Fig. 4G-J).

Moreover, treatment with FB23-2, a demethylation inhibitor targeting FTO, was also performed simultaneously with GnRH treatment. RT-qPCR results showed that treatment with FB23-2 was able to significantly reduce the promotion effect of GnRH on the mRNA expression of *Fshb*, *Lhb*, *Fto*, and *Foxp2* (Additional file 1: Fig. S7A, B). WB results showed that the treatment with FB23-2 was able to significantly decrease the promotion of FTO and FOXP2 protein expression by GnRH (Additional file 1: Fig. S7C-E). The above results indicated that FTO was able to promote gonadotropin synthesis and secretion by promoting FOXP2 expression after stimulation with GnRH.

### FTO-mediated de-m^6^A modification enhances *Foxp2* mRNA stability

**Fig. 5:**
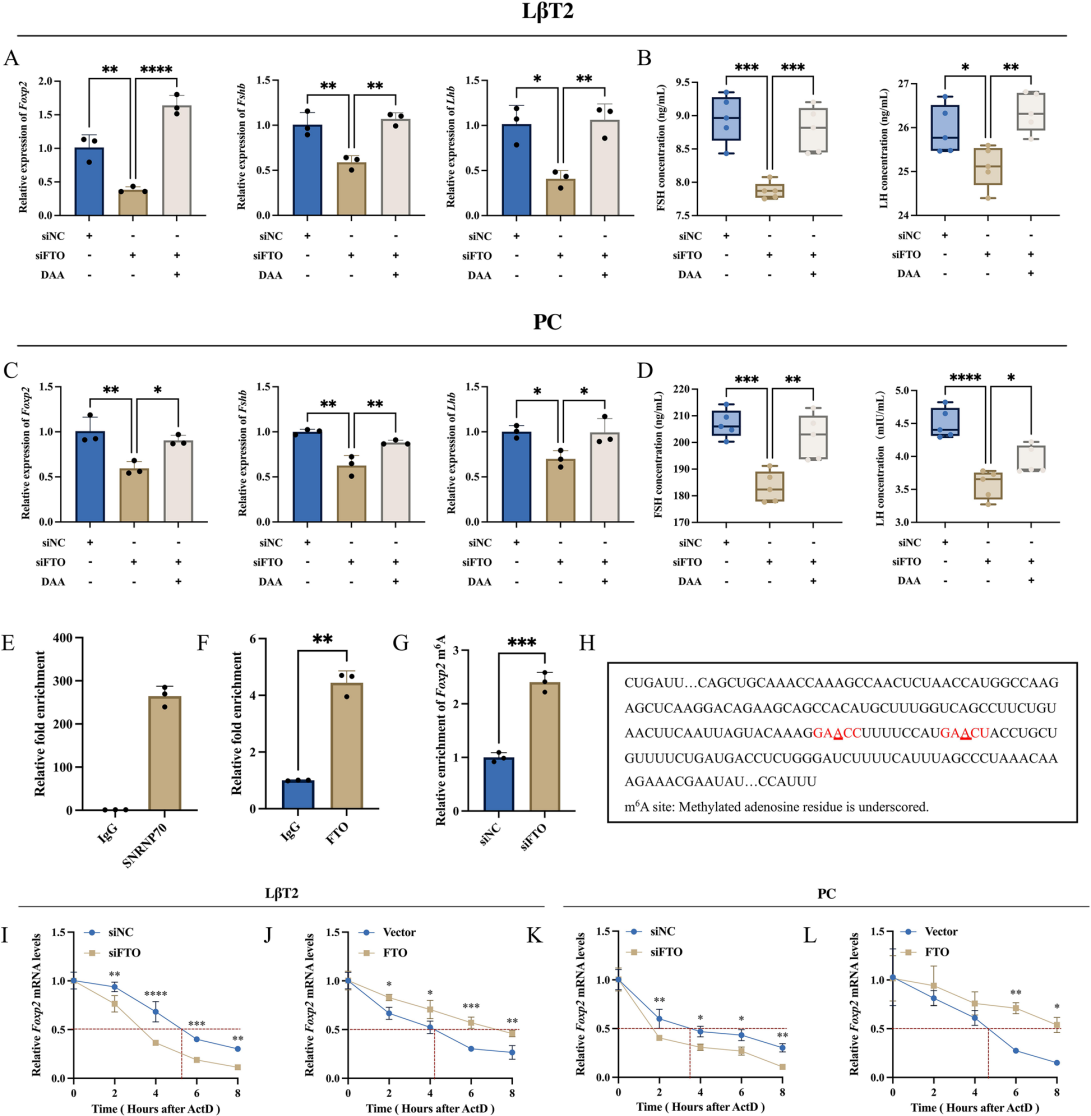
FTO-mediated de-m^6^A modification enhances *Foxp2* mRNA stability. **A**-**D** FTO siRNA or siNC were transfected with or without DAA (25 μM). The expression of *Foxp2*, *Fshb* and *Lhb* mRNA was assayed by RT-qPCR in LβT2 cells (**A**, *n* = 3) and primary rat adenohypophysis cells (**C**, *n* = 3). The secretion of FSH and LH was assayed by ELISA in LβT2 cells (**B**, *n* = 5) and primary rat adenohypophysis cells (**D**, *n* = 5). **E** Negative and positive controls for RIP (*n* = 3). **F** RIP to verify the interaction of FTO protein with *Foxp2* mRNA (*n* = 3). **G** MeRIP-qPCR to detect the enrichment of m^6^A modification on *Foxp2* mRNA after knockdown FTO (*n* = 3). **H** m.^6^A single-base modification sites on *Foxp2* mRNA that may be regulated by FTO. **I**-**L** *Foxp2* mRNA stability assay in LβT2 cells transfected with FTO siRNA (**I**) or FTO overexpression plasmid (**J**). *Foxp2* mRNA stability assay in primary rat adenohypophysis cells transfected with FTO siRNA (**K**) or FTO overexpression plasmid (**L**). *, *P* < 0.05; **, *P* < 0.01; ***, *P* < 0.001; ****, *P* < 0.0001

Treatment with the m^6^A modification inhibitor DAA was performed simultaneously with FTO siRNA transfection to investigate whether FTO regulates *Foxp2* mRNA expression and gonadotropin synthesis and secretion by mediating de-m^6^A modification. RT-qPCR results showed that DAA could significantly reverse the reduction in the mRNA expression levels of *Fshb*, *Lhb*, and *Foxp2* induced by the knockdown of FTO (Fig. 5A, C). ELISA results showed that DAA was able to significantly reverse the reduction in FSH and LH secretion levels caused by the knockdown of FTO (Fig. 5B, D). This revealed that the involvement of m^6^A modification was required in the regulation of *Foxp2* mRNA expression and gonadotropin synthesis and secretion by FTO.

Furthermore, RIP confirmed the direct interaction between FTO protein and *Foxp2* mRNA (Fig. 5E, F). Two very close m^6^A single-base modification sites, which were located in the 3' UTR and possibly regulated by FTO, were screened on *Foxp2* mRNA by combining them with the SRAMP database [36] and the previous m^6^A-seq data (Fig. 5H). MeRIP-qPCR results showed that the knockdown of FTO was able to significantly promote the m^6^A modification enrichment level on these two sites on *Foxp2* mRNA (Fig. 5G). The results of the RNA stability assay showed that knockdown of FTO resulted in faster decay and a shorter half-life of *Foxp2* mRNA (Fig. 5I, K); overexpression of FTO showed the opposite result (Fig. 5 J, L). The above results indicated that FTO-mediated de-m^6^A modification could enhance *Foxp2* mRNA stability.

### YTHDF3 inhibits gonadotropin synthesis and secretion via recognizing m^6^A modifications on *Foxp2* to promote its mRNA degradation

**Fig. 6:**
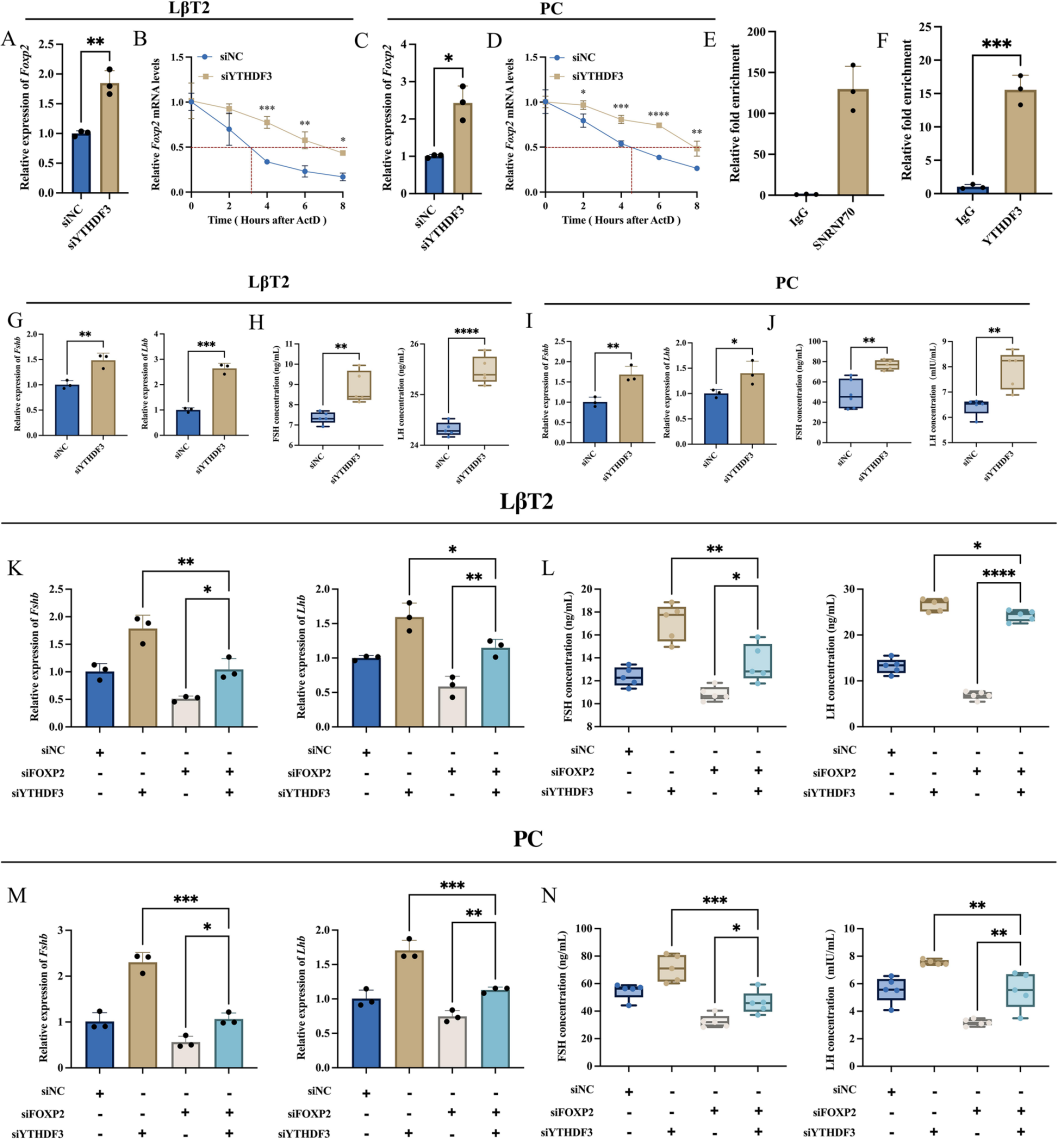
YTHDF3 inhibits gonadotropin synthesis and secretion via promoting *Foxp2* mRNA degradation. **A** RT-qPCR analysis *Foxp2* mRNA expression in LβT2 cells transfected with siNC (siNC group) or YTHDF3 siRNA (siYTHDF3 group) (*n* = 3). **B** *Foxp2* mRNA stability assay in LβT2 cells transfected with YTHDF3 siRNA. **C** RT-qPCR analysis *Foxp2* mRNA expression in primary rat adenohypophysis cells transfected with siNC (siNC group) or YTHDF3 siRNA (siYTHDF3 group) (*n* = 3). **D** *Foxp2* mRNA stability assay in primary rat adenohypophysis cells transfected with YTHDF3 siRNA. **E** Negative and positive controls for RIP (*n* = 3). **F** RIP to verify the interaction of YTHDF3 protein with *Foxp2* mRNA (*n* = 3). **G**-**J** RT-qPCR analysis *Fshb* and *Lhb* mRNA expression transfected with siNC (siNC group) or YTHDF3 siRNA (siYTHDF3 group) in LβT2 cells (**G**, *n* = 3) and primary rat adenohypophysis cells (**I**, *n* = 3), respectively. ELISA analysis the secretion levels of FSH and LH transfected with siNC (siNC group) or YTHDF3 siRNA (siYTHDF3 group) in LβT2 cells (**H**, *n* = 5) and primary rat adenohypophysis cells (**J**, *n* = 5), respectively. **K**-**N** FOXP2 siRNA was transfected with or without YTHDF3 siRNA. The expression of *Fshb* and *Lhb* mRNA was assayed by RT-qPCR in LβT2 cells (**K**, *n* = 3) and primary rat adenohypophysis cells (**M**, *n* = 3). The secretion of FSH and LH was assayed by ELISA in LβT2 cells (**L**, *n* = 5) and primary rat adenohypophysis cells (**N**, *n* = 5). *, *P* < 0.05; **, *P* < 0.01; ***, *P* < 0.001; ****, *P* < 0.0001

The RM2Target database[37] was used to mine m^6^A readers that may be involved in regulating *Foxp2* mRNA stability. YTHDF3 was found to be able to interact with *Foxp2* mRNA and there was a negative regulatory relationship between them. It was hypothesized that YTHDF3 might be involved in regulating m^6^A modification-mediated *Foxp2* mRNA destabilization. To test this scientific hypothesis, siRNAs of YTHDF3 were transfected into LβT2 cells and primary rat adenohypophysis cells. The results showed that the expression of *Foxp2* mRNA was significantly increased (Fig. 6A, C), the decay of *Foxp2* mRNA was slower and the half-life was longer after the knockdown of YTHDF3 (Fig. 6B, D). RIP confirmed the direct interaction between YTHDF3 protein and *Foxp2* mRNA (Fig. 6E, F). The above results indicated that YTHDF3 was able to interact with *Foxp2* mRNA and promote its degradation, which in turn inhibited FOXP2 expression.

The changes in gonadotropin synthesis and secretion after transfection of YTHDF3 siRNA were further detected. RT-qPCR results showed that the knockdown of YTHDF3 significantly promoted the mRNA expression of *Fshb* and *Lhb* (Fig. 6G, I). ELISA results showed that the knockdown of YTHDF3 significantly promoted the secretion of FSH and LH (Fig. 6H, J). Rescue experiments were performed, in which FOXP2 siRNA was cotransfected with YTHDF3 siRNA. RT-qPCR and ELISA results showed that cotransfection was able to reverse the regulatory effects of YTHDF3 and FOXP2 on gonadotropin synthesis and secretion (Fig. 6K-N). These results confirmed that YTHDF3 recognized the m^6^A modification on *Foxp2* mRNA and promoted its degradation, thereby inhibiting gonadotropin synthesis and secretion. In addition, the above molecular mechanisms were further verified using heterozygous *Fto* knockout mice (*Fto*^+/−^). RT-qPCR results showed that the expression levels of *Fto*, *Foxp2*, *Fshb,* and *Lhb* mRNA were significantly reduced in the pituitary tissues of *Fto*^+/−^ mice (Additional file 1: Fig. S8A-D). ELISA results also showed that FSH and LH secretion levels were significantly reduced (Additional file 1: Fig. S8E, F). Collectively, FTO-mediated m^6^A modification can regulate FOXP2 expression by affecting YTHDF3 binding, which subsequently promotes gonadotropin synthesis and secretion.

## Discussion

**Fig. 7:**
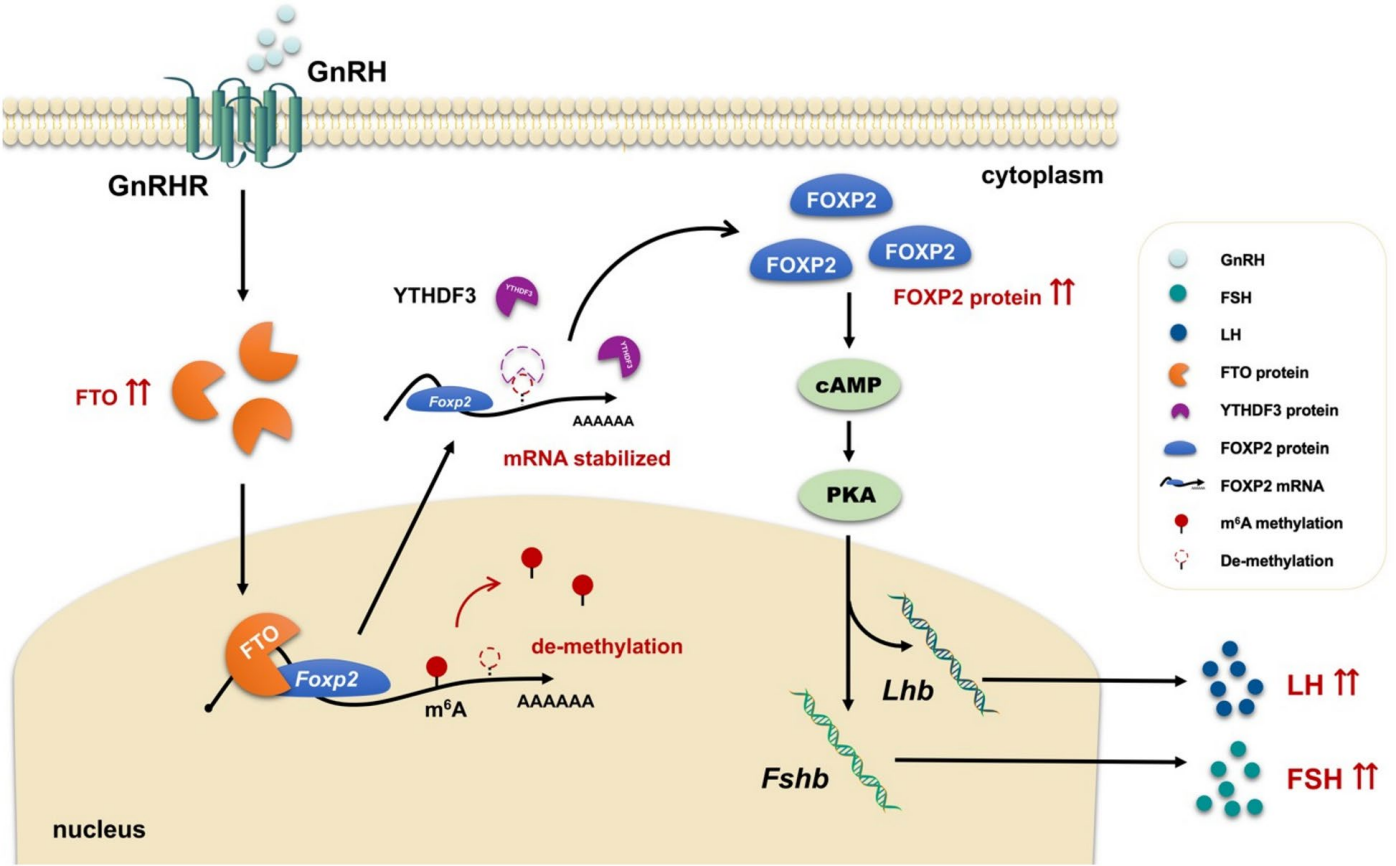
A proposed model of FTO-promoted gonadotropin synthesis and secretion. Under GnRH stimulation, the m^6^A eraser FTO is upregulated. Highly expressed FTO enters the nucleus and binds to *Foxp2* mRNA, reducing m^6^A modification at the 3’UTR regions. After *Foxp2* mRNA exits the nucleus, the lack of m^6^A modification prevents the m^6^A reader YTHDF3 from binding to it, resulting in increased stability and up-regulation of *Foxp2* mRNA expression, which activates the cAMP/PKA signaling pathway to promote the gonadotropins synthesis and secretion

The pituitary, as one of the endocrine organs, secretes a variety of reproductive hormones and plays an important pivotal role in the regulation of mammalian reproductive development through HPGA [38]. The mechanism by which GnRH affects gonadotropin synthesis and secretion remains an important question in the study of animal reproduction. Accumulating evidence suggests that m^6^A modifications participate in the regulation of animal reproductive processes such as spermatogenesis, oocyte maturation, and early embryonic development [39, 40]. The normal collaboration of m^6^A modification-related enzymes and the dynamic balance of m^6^A modification need to maintain normal reproductive activities in animals. However, little is known about the role of m^6^A modifications in the regulation of gonadotropin synthesis and secretion. The results of this study showed that the expression level of the demethylase FTO was significantly upregulated in the GnRH-treated adenohypophysis. Highly expressed FTO erases the m^6^A modification of the 3' UTR of the FOXP2 transcript upon GnRH stimulation, preventing the recognition and binding of YTHDF3 and thus promoting FOXP2 translation. The biosynthesis of FOXP2 activates the cAMP/PKA signaling pathway, which promotes gonadotropin synthesis and secretion (Fig. 7).

In addition to activating specific transcription factors during the regulation of gonadotropin synthesis and secretion, GnRH is able to promote the transcription of target genes through a variety of epigenetic regulatory mechanisms [41]. The discovery of novel epigenetic mechanisms, including m^6^A modification, suggests that there is still an enormous uncharted space in the existing regulatory network of gonadotropin synthesis and secretion that needs to be explored. According to our data, we proposed that FTO is a GnRH-stimulated responsive gene because adenohypophyseal FTO is as significantly upregulated as gonadotropin-related genes when cells are subjected to GnRH treatment. The level of m^6^A modification in gonadotropin cells was also significantly reduced by GnRH stimulation. However, our data did not elucidate the reason why GnRH promotes FTO expression. The exact mechanism by which GnRH affects the enzymatic activity of m^6^A demethylases needs to be further explored. Furthermore, cotreatment with FB23-2, a demethylation inhibitor targeting FTO, confirmed that inhibition of FTO blocked the promotion of downstream gene expression by GnRH. Our results also suggested that FTO was capable of promoting gonadotropin synthesis and secretion. Nevertheless, the pre-m^6^A-seq results showed that the m^6^A modifications on *Fshb* and *Lhb* mRNA did not show significant differences due to GnRH stimulation. In this way, FTO is not involved in the regulation of gonadotropin synthesis and secretion by directly regulating the m^6^A modifications on *Fshb* and *Lhb* mRNA, and there may be a mediator involved in this process.

Interestingly, it was found that the *Foxp2* gene was highly expressed in our preconstructed differential gene expression profile of GnRH-treated pituitary tissues; and the present study also confirmed the ability of GnRH to promote the expression of FOXP2 in gonadotropin cells. FOXP2, the first gene found to be associated with voice and speech disorders, has been the subject of much of the initial functional research centered on its drive for neurological evolution, developmental disorders, and neurodegenerative diseases [42]. In 2018, FOXP2 was shown for the first time to be expressed in the adenohypophysis of adult male mice with a predisposition for gonadotropin cell population-specific enrichment of expression [43]. This finding not only provides a new cellular fractionation marker for future studies on the pituitary function but also suggests that FOXP2 is likely to play some unknown biological functions in gonadotropin cells. In addition, it has been reported to respond to androgen signaling and thus mediate sex differences in speech and vocalization [44]. Thus, it is reasonable to speculate that FOXP2, which is specifically enriched in gonadotropin cells, plays a potential role in the cellular response to GnRH signaling and regulating gonadotropin synthesis and secretion. In our study, we found that FOXP2 does indeed have a promotional effect on the synthesis and secretion of both FSH and LH. FOXP2, as a transcription factor, has dual-sided effects on the regulation of downstream target gene expression. On the one hand, FOXP2 is able to function as a transcriptional repressor by directly binding to the regulatory sequences of genes such as *Cntnap2* [45], *Srpx2* [46], *Met* [47], and *Disc1* [48], which in turn represses gene expression. On the other hand, FOXP2 can also promote gene transcription [49]. Therefore, the possible molecular mechanisms by which FOXP2 regulates gonadotropin synthesis and secretion are explored further here in terms of transcriptional regulation, cell apoptosis, and pathway activation. Inconsistent with the expected results, FOXP2 did not affect gonadotropin cell apoptosis and there was no interaction between its protein and *Fshb* DNA. FOXP2 did not act as a transcription factor by affecting gene transcription but rather by activating the cAMP/PKA signaling pathway to regulate the expression of *Fshb* and *Lhb* mRNA, which in turn has a promotional effect on gonadotropin synthesis and secretion.

An increasing number of posttranscriptional regulatory mechanisms regulating FOXP2 expression have been demonstrated in recent years. The discovery of various non-coding RNAs, such as miR-134-5p [50], miR-628-5p [51], lncRNA XIST [52], lncRNA MALAT1 [53], and circ-SHKBP1 [54] has also enriched the regulatory network of FOXP2 expression. Here, the level of m^6^A modification in the 3' UTR of *Foxp2* mRNA was significantly reduced in the GnRH-treated adenohypophysis. FOXP2 expression was positively regulated by FTO. Validation of the regulatory relationship alone, certainly, did not suggest that m^6^A modification plays a critical role since the function of FTO is not limited to regulating m^6^A modification. Therefore, the present study clarified the necessity of m^6^A modification in the regulation of FOXP2 expression and gonadotropin synthesis and secretion by FTO through m^6^A modification inhibitor treatment experiments. It has been well established that DAA is able to block the insertion of the m^6^A motif into the mRNA substrate by inhibiting SAH hydrolysis [55]. In this study, the result that DAA cotreatment reverted to knockdown of FTO-induced changes in downstream gene expression suggested that FTO regulated the expression of FOXP2 by mediating m^6^A modification, which in turn affected the synthesis and secretion of gonadotropins. Wei et al. recently found that FTO can mediate the demethylation of m^6^A modification on *Line1* mRNA in mouse embryonic stem cells and maintain *Line1* mRNA abundance [56]. Similarly, potential m^6^A modification sites on *Foxp2* mRNA were analyzed in this study. Based on our studies in previous studies, it has been determined that m^6^A modification of the 3' UTR of pituitary *Foxp2* mRNA was reduced after GnRH treatment. At the same time, we have predicted several sites located in the 3' UTR region of *Foxp2* mRNA with high scores by the SRMAP database. Combining the specific site locations getting from the above two efforts, sites 3367 and 3380, which are located in the 3' UTR and very close to each other, were selected as candidate sites finally. RIP and MeRIP-qPCR confirmed that FTO was able to interact with *Foxp2* mRNA and mediate the de-m^6^A modification of these two single-base sites. The regulation of *Foxp2* mRNA stability by FTO was analyzed by the actinomycin D assay, and it was found that FTO-mediated de-m^6^A modification enhanced *Foxp2* mRNA stability.

The m^6^A modification exerts its biological function by the "m^6^A switch" mechanism [57]. This suggests that the binding of different "readers" determines the direction of the different fates of m^6^A-modified mRNA processing, translocation, translation, and decay. For example, FTO-mediated reduction in m^6^A modification on *Atg5* and *Atg7* mRNA can reduce the binding of YTHDF2, maintain the stability of *Atg5* and *Atg7* mRNA while promoting their translation, and thus regulate normal cellular autophagy and adipogenesis in the body [58]. This makes it reasonable to speculate in this study that the reduction in binding of a particular methylation-recognizing enzyme has contributed to a more stable *Foxp2* mRNA. It has been reported that m^6^A-labeled mRNAs tend to be more inclined to be in an unstable state [59]. However, this tendency is no longer solely due to YTHDF2-mediated mRNA decay according to recent research. Zaccara et al. presented more evidence that YTHDF1, YTHDF2, and YTHDF3 proteins have similar rather than different functions. All three YTHDF proteins redundantly induce degradation of the same mRNAs, and all m^6^A-modified mRNAs are subjected to the combined action of these proteins in proportion to the number of m^6^A-modified sites [60]. The proposal of this new functionally unified model of m^6^A modification provides a reference for the exploration of the m^6^A modification mechanism while having a great impact on the existing mechanism model. In this study, we mined the methylation-recognizing enzymes that may regulate the stability of *Foxp2* mRNA and found that YTHDF3 was able to interact with *Foxp2* mRNA, and there was a negative regulatory relationship between the two. It was further demonstrated that YTHDF3 could play a role in the regulation of gonadotropin synthesis and secretion by decreasing the stability of *Foxp2* mRNA and its expression level.

Heterozygous *Fto* knockout mice were utilized to more rigorously validate the molecular mechanisms revealed by the study. After ensuring that FTO expression was significantly reduced, the expression levels of *Foxp2*, *Fshb,* and *Lhb* mRNA were found to be reduced at different levels in the pituitary of *Fto*^+/−^ mice. *Fto*^+/−^ mice also showed lower levels of gonadotropin secretion than wild-type mice. However, there are obvious limitations to this result. Although some studies have reported that the construction of pure *Fto* global knockout mice is possible, we did not obtain any pure mice during our breeding [61]. This may be due to differences in acquisition methods. Therefore, we can only use heterozygous mice for the time being to perform a simple verification of the regulatory relationship between the above mechanisms. In the future, it will also continue to try to construct pure *Fto* global knockout mice or conditional pituitary knockout mice to better explore the function of FTO and m^6^A modifications in the pituitary. In addition, another limitation of the present study is the selective synthesis of FSH and LH produced by different frequencies of GnRH stimulation. It is now generally accepted that GnRH pulse stimulation at a low frequency (approximately 120-min interval) preferentially promotes the expression of the *Fshb* and thus FSH secretion, while GnRH pulse stimulation at a high frequency (approximately 30-min interval) preferentially promotes the expression of the *Lhb* and thus LH secretion [62]. This different physiological effect produced by different frequencies leads to the mechanism of selective synthesis of FSH and LH in mammals. However, we did not further explore whether there are differences in the regulatory effects of GnRH treatment on FTO expression, FOXP2 expression, and m^6^A modification changes at different frequencies. We will further try to explore the molecular mechanism of the different physiological effects induced by different frequencies of GnRH by the cell perfusion culture system in the future.

Overall, this study revealed that FTO was able to reduce the m^6^A modification on *Foxp2* mRNA, prevent YTHDF3-mediated *Foxp2* mRNA decay, and increase *Foxp2* mRNA stability and expression, which in turn promoted the synthesis and secretion of gonadotropins. This study not only reveals the functional importance of m^6^A modification in gonadotropin synthesis and secretion but also provides a theoretical basis for the improvement of artificial reproduction control techniques, such as estrus control, follicle induction and development, and supernumerary ovulation.

## Conclusions

GnRH promotes the expression of FTO in gonadotropin cells. Highly expressed FTO enters the nucleus and binds to *Foxp2* mRNA, mediating de-m^6^A modification on it. After *Foxp2* mRNA exits the nucleus, the lack of m^6^A modification on *Foxp2* mRNA prevents YTHDF3 from binding to *Foxp2* mRNA, resulting in enhanced stability of *Foxp2* mRNA and up-regulation of FOXP2 expression, which promotes gonadotropin synthesis and secretion through activation of the cAMP/PKA signaling pathway.

## Methods

### Ethics approval

The experimental protocol of this study was approved by the Institutional Animal Care and Use Committee of Jilin University and was conducted in strict accordance with the Laboratory Animal Guideline for ethical review of animal welfare. All animal experiments were performed in compliance with the welfare of laboratory animals (Permit Number: SY20200508 and SY202302029).

### Animals & tissue collection

The 8-week-old SPF-grade male Sprague–Dawley (SD) rats used in the study were purchased from Liaoning Changsheng Biotechnology Co, Ltd. *Fto* knockout mice were constructed from by Cyagen Biosciences (Suzhou) Inc. The results were reported for 8-week-old male mice in the same genetic background (C57BL6/J). All rats and mice were kept in barrier facilities for SPF laboratory animals for experimental research under a 12-h/12-h light/dark cycle with unrestricted access to food and water.

Rats were randomly divided into a GnRH-treated group and a control group. Two intraperitoneal injections of GnRH were administered to the GnRH-treated group, with each rat receiving 1 mL of 0.2 μg/mL injectable gonadorelin (a GnRH analog, Ningbo Second Hormone Factory, Ningbo, China) per injection. The interval between the two injections was 2 h. The control group was injected with an equal amount of saline. Rats were euthanized within 10 min after the second injection using the automated CO_2_ delivery system of the CO_2_ euthanasia machine, and pituitary tissue was collected. After stripping the neurohypophysis, rat adenohypophysis tissue was collected for subsequent experiments.

### Cell culture

Primary rat adenohypophysis cells: Rat adenohypophysis tissue from 8-week-old male SD rats was obtained after stripping the neurohypophysis and washed with prechilled PBS (MA0015, Meilunbio, Dalian, China). The cleaned adenohypophysis tissue was cut into the smallest possible pieces and digested at 37 °C for 90 min in 2.5 g/L collagenase type I (17,100,017, Gibco, USA). The tissue was gently blown for homogenization, and the tissue suspension was filtered using a 70 μm cell sieve (15–1070, Biolgix, Shanghai, China). The filtered tissue suspension was centrifuged at 200 × g for 10 min to obtain cell precipitates. The cell precipitate was resuspended in DMEM/F12 medium (01–170-1A, BI, Israel) containing 10% fetal bovine serum (S711-001S, Lonsera, Uruguay) and 1% penicillin/streptomycin (15,140,122, Gibco, USA). The obtained primary rat adenohypophysis cells were cultured at 37 °C under 5% CO_2_. All cells used in the study were routinely tested for contamination.

LβT2 cells: LβT2 cells were used with permission from Prof. Pamela L. Mellon (University of California, USA) and gifted by Prof. Jing Liu (Zhejiang University, China). The required cell culture dishes or plates were treated with poly-L-lysine (P4707, Sigma, USA) in advance to ensure proper apposition of LβT2 cells. The cells were maintained in high glucose DMEM medium (01–052-1A, BI, Israel) containing 10% fetal bovine serum (S711-001S, Lonsera, Uruguay) and 1% penicillin/streptomycin (15,140,122, Gibco, USA) at 37 °C under 5% CO_2_. LβT2 cells were passaged with trypsin–EDTA solution containing 0.05% trypsin and 0.02% EDTA (C0204, Beyotime, Shanghai, China) due to being sensitive to over-trypsinization and excessively sparse plating. All cells used in the study were routinely tested for contamination.

### Cell treatment

GnRH (HY-P0292), 2',5'-Dideoxyadenosine (DDA, HY-135878), H89 (HY-15979), 8-Bromo-cAMP (HY-12306), FB23-2 (HY-127103), and 3-Deazaadenosine (DAA, HY-W013332) used to treat the cells were purchased from MedChemExpress. The detailed treatment duration and concentrations were as follows: GnRH, 8 h, 100 nM (rat adenoidal primary cells), 10 nM (LβT2 cells); DDA, 24 h, 10 μM; H89, 24 h, 10 μM; 8-Bromo-cAMP, 24 h, 500 μM; FB23-2, 8 h, 20 μM; and DAA, 24 h, 25 μM.

### Cell transfection

LβT2 cells and primary rat adenohypophysis cells were cultured in six-well plates using the complete medium without penicillin/streptomycin, and the cell density was increased to 40%-60% for cell transfection. Transfection experiments of small interfering RNA (siRNA) and overexpression plasmids were performed using the universal transfection reagent Trans-Mate (Jtsbio, Wuhan, China). According to the manufacturer's protocol, 160 pmol of siRNA or 2 μg of overexpression plasmid with 6 μL of Trans-Mate was added to each well. The change in mRNA expression could be detected after 24 h, and the change in protein expression could be detected after 48 h. The transfection efficiency was examined by RT-qPCR, and the sample with the best efficiency was selected for subsequent studies (Additional file 1: Fig. S9). All siRNAs were constructed by Jtsbio (Wuhan, China), and all overexpression plasmids were constructed by GenePharma (Suzhou, China). The sequences of siRNAs used in the study are detailed in the additional file (Additional file 2: Table S1).

### RNA extraction and RT-qPCR

Total RNA was extracted using the SevenFast^®^ Total RNA Extraction Kit (SM132, Seven Biotech, Beijing, China). cDNA was synthesized from the extracted RNA samples using the MonScript™ RTIII All-in-One Mix with dsDNase (MR05101, Monad, Suzhou, China), and RT-qPCR was performed using the MonAmp™ ChemoHS qPCR Mix (MQ00401, Monad, Suzhou, China). All procedures were performed according to the manufacturer's protocol and the primers used for the study are detailed in the additional file (Additional file 2: Table S2, S3). The relative expression of target genes was analyzed by the 2-ΔΔCT method using *Gapdh* as the reference gene.

#### ELISA

The levels of FSH and LH in the culture supernatants of primary rat adenohypophysis cells before and after treatment were measured using a rat FSH ELISA kit (E-EL-R0391c, Elabscience, Wuhan, China) and a rat LH ELISA kit (E-EL-R0026c, Elabscience, Wuhan, China). The levels of FSH and LH in the culture supernatants of LβT2 cells before and after treatment were measured using the Mouse FSH ELISA Kit (E-EL-M0511c, Elabscience, Wuhan, China) and the Mouse LH ELISA Kit (E-EL-M3053, Elabscience, Wuhan, China).

### Protein extraction and western blotting

Protein was extracted using high-performance RIPA lysis buffer containing 1% PMSF (PC101, Epizyme, Shanghai, China). The extracted protein concentrations were measured using the BCA Protein Assay Kit (P0010, Beyotime, Shanghai, China). A 1/4 volume of 5 × protein loading buffer (LT101, Epizyme, Shanghai, China) was added to the extracted protein sample, and the sample was mixed well and denatured at 95 °C for 10 min. The SDS‒PAGE gel for separation was prepared using the PAGE Gel Fast Preparation Kit (PG112, Epizyme, Shanghai, China) according to the manufacturer’s protocol. Proteins were transferred from the gel to polyvinylidene difluoride (PVDF) membranes (IPVH00010, Millipore, USA) using a Mini Trans-Blot™ system (Bio-Rad, USA). The PVDF membrane was incubated with proteins sequentially with 1 × Protein Free Rapid Blocking Buffer (30 min at room temperature on a shaker, PS108P, Epizyme, Shanghai, China), primary antibody (1:1000 dilution, overnight at 4 °C), and HRP-conjugated secondary antibody (1:3000 dilution, 1 h at room temperature on a shaker), followed by washing five times with 1 × TBS/Tween buffer (PS103, Epizyme, Shanghai, China) after incubation with antibody dilution (6 min at room temperature on a shaker). The antibodies used were as follows: FTO antibody (ab280081, Abcam, UK); FOXP2 antibody (AF5385, Affinity, USA); cathelicidin antibody (DF7741, Affinity, USA); PKA antibody (AF7746, Affinity, USA); phospho-PKA antibody (AF7246, Affinity, USA); and HRP-linked anti-rabbit IgG antibody (7074, Cell Signaling Technology, USA). The on-membrane proteins were detected using an Omni-ECL™ Femto Light Chemiluminescence Kit (SQ201, Epizyme, Shanghai, China). The bands were analyzed using ImageJ for statistical analysis. Raw blot images are available in Additional file 3.

### Yeast one-hybrid assay

The DNA sequence of the *Fshb* gene was inserted into the pAbAi vector. The recombinant plasmid pAbAi-*Fshb* was linearized using BstBI restriction endonuclease (R0519, NEB, USA). The enzyme cleavage system is detailed in the additional file (Additional file 2: Table S4). Integration of pAbAi-*Fshb* into the Y1Hgold yeast genome after successful linearization was confirmed by PCR. A self-activation assay of Y1Hgold [pAbAi-*Fshb*] was performed after successful integration by PCR. The FOXP2 overexpression plasmid was transferred into Y1Hgold[pAbAi-*Fshb*] in the absence of self-activation or with slight self-activation. The interactions between FOXP2 and *Fshb* DNA were explored by point-to-point validation and point-plate validation experiments.

### Apoptosis assay

LβT2 cells and primary rat adenohypophysis cells were transfected with the FOXP2 siRNA, FOXP2 overexpression plasmid, or corresponding controls. Flow analysis was performed using an Annexin V-FITC/PI apoptosis kit (AP101, Multisciences, Hangzhou, China) according to the manufacturer’s protocol, and apoptosis of cells was analyzed according to the flow cytometry results.

### Intracellular cAMP level assay

LβT2 cells and primary rat adenohypophysis cells were transfected with the FOXP2 overexpression plasmid or vector. The intracellular cAMP level was detected by a cAMP Assay Kit (ab65355, Abcam, UK) according to the manufacturer’s protocol. The amount of cAMP was determined by measuring the absorbance at OD 450 nm, and the level of signal was compared to a standard curve for free cAMP to obtain the intracellular cAMP level.

### Immunohistochemistry

Rat adenohypophysis tissues were fixed in 4% paraformaldehyde (MA0192, Meilunbio, Dalian, China) for 48 h and then made into paraffin sections. After dewaxing and rehydration, the sections were antigenically repaired using EDTA antigen retrieval solution (P0084, Beyotime, Shanghai, China). The samples were sequentially incubated in blocking solution (incubated for 30 min at room temperature), primary antibody (FTO antibody 1:2000 dilution, FOXP2 antibody 1:200 dilution, overnight at 4 °C), and secondary antibody (1:500 dilution, incubated for 1 h at room temperature away from light). The antibodies used were as follows: FTO antibody (ab280081, Abcam, UK); FOXP2 antibody (20,529–1-AP, Proteintech, USA); and anti-rabbit IgG (H + L) antibody (5220–0336, SeraCare, USA). Both antibody incubations were followed by washing three times with PBS (5 min at room temperature). The nucleus was restained with hematoxylin staining solution (C0107, Beyotime, Shanghai, China) after development using a DAB horseradish peroxidase color development kit (P0203, Beyotime, Shanghai, China). The sections were dehydrated and sealed. Images were visualized and collected using an Olympus fluorescence microscope. Three randomly selected images from the immunohistochemistry results were analyzed for positive reaction areas using ImageJ for statistical analysis.

### Immunofluorescence

LβT2 cells treated in 12-well plates were fixed at room temperature for 10 min with 500 μL of 4% paraformaldehyde per well, followed by washing three times with precooled PBS (5 min at room temperature). Cells were permeabilized with 500 μL of PBS containing 0.25% Triton X-100 (T8200, Solarbio, Beijing, China) per well for 10 min at room temperature, followed by washing three times with precooled PBS (5 min at room temperature). The samples were incubated with blocking solution (30 min at room temperature), primary antibody (FTO antibody 1:50 dilution, overnight at 4 °C), and secondary antibody (1:500 dilution, incubate for 1 h at room temperature away from light). The antibodies used were as follows: FTO antibody (ab280081, Abcam, UK) and IFKine™ Green Donkey Anti-Rabbit IgG (A24221, Abbkine, USA). Both antibody incubations were followed by washing three times with PBS (5 min at room temperature protected from light). The nucleus was stained and sealed with an anti-fluorescence quenching sealer containing DAPI (P0131, Beyotime, Shanghai, China). The coverslips were then mounted onto slides, and images were visualized and collected using an Olympus fluorescence microscope.

### RNA m^6^A quantification

The total RNA m^6^A modification level in LβT2 cells after GnRH treatment was detected using the m^6^A RNA Methylation Assay Kit (ab185912, Abcam, UK) according to the manufacturer’s protocol.

### RNA stability assay

LβT2 cells and primary rat adenohypophysis cells were treated with 5 μg/mL actinomycin D (HY-17559, MedChemExpress, USA) and collected at indicated time points (0 h, 2 h, 4 h, 6 h and 8 h). The stability of the mRNA was detected by RT-qPCR after extracting the total RNA.

### RNA immunoprecipitation

RNA immunoprecipitation (RIP) assays were performed using the Magna RIP® RNA Binding Protein Immunoprecipitation Kit (17–700, Millipore, USA). For the preparation of the target protein beads, 5 μg of FTO antibody (27,226–1-AP, Proteintech, USA) and 5 μg of YTHDF3 antibody (ab220161, Abcam, UK) were added. For the preparation of negative control beads, 5 μg of IgG antibody was added, and for the preparation of positive control beads, 5 μg of SNRNP70 antibody was added. The remaining procedure was performed strictly according to the manufacturer's detailed protocol. The obtained RNA was purified using a MagenTM Hipure Serum/plasma miRNA kit (R4317, Magen, Guangzhou, China). The enrichment of target proteins was measured with RT-qPCR on purified RNA. The primers used for the study are detailed in the additional file (Additional file 2: Table S5).

### MeRIP-qPCR

LβT2 cells were cultured in 15 cm cell culture dishes and transfected with FTO siRNA or the corresponding control. RNA from the transfected cells was extracted (the mass of total RNA was not less than 50 μg) and cleaved into fragments of approximately 200 bp. Furthermore, RNA methylation immunoprecipitation experiments were performed using the riboMeRIPTM m^6^A Transcriptome Profiling Kit (R11096, RiboBio, Guangzhou, China) according to the detailed experimental protocol. The obtained RNA was purified using a MagenTM Hipure Serum/plasma miRNA kit. The enrichment of m^6^A modifications was measured with RT-qPCR on purified RNA. The primers used for the study are detailed in the additional file (Additional file 2: Table S5).

### Bioinformatic analysis

Pre-pituitary single-cell transcriptomics data (PRJNA1084893, NCBI BioProject) were mined using the R package (V4.1.1). The bubble plots were drawn to demonstrate the expression of *Foxp2*, *Fshb*, and *Lhb* mRNA in various cell populations of the pituitary by FlexDotPlot. IGV peak plot of m^6^A modification on *Foxp2* mRNA of GnRH-treated pituitary was constructed based on pre-m^6^A-seq (PRJNA792448, SRA database)[35].

### Statistics and reproducibility

Data were analyzed and figures were generated using GraphPad Prism 9 (Prism for Mac OS X, GraphPad Software Inc., San Diego, California). The data are presented as the mean ± standard deviation (mean ± SD) for at least 2 repeated individual experiments. The t-test was used to statistically analyze the data in both groups and one-way ANOVA was used to statistically analyze the data in multiple groups. Differences were considered statistically significant at *P* < 0.05. *P < 0.05, **P < 0.01, ***P < 0.001 and ****, *P* < 0.0001 denote the significance thresholds.

### Supplementary Information

**Additional file 1:** **Fig. S1.** GnRH promotes rat gonadotropin synthesis and secretion. **Fig. S2.** GnRH promotes gonadotropin synthesis and secretion and FTO expression in LβT2 cells and primary rat adenohypophysis cells. **Fig. S3.** Expression distribution of *Foxp2* mRNA in rat pituitary and the distribution of its upper m^6^A modifications. **Fig. S4.** Validation of FOXP2 interactions with *Fshb* DNA. **Fig. S5.** Apoptosis of LβT2 cells and primary rat adenohypophysis cells after FOXP2 knockdown or overexpress. **Fig. S6.** FOXP2 regulates gonadotropin synthesis and secretion via activating the cAMP/PKA signaling pathway in primary rat adenohypophysis cells. **Fig. S7.** GnRH stimulation is dependent on FTO expression. **Fig. S8.** Decreased gonadotropin synthesis and secretion in *Fto*^+/−^ mice. **Fig. S9.** Transfection efficiency assay of siRNAs and plasmids by RT-qPCR.

**Additional file 2:** **Table S1.** Sequences of siRNAs used in the study. **Table S2.** Mouse primers used in the study. **Table S3**. Rat primers used in the study. **Table S4.** Enzyme-cutting system for plasmid linearization. **Table S5. **Primers used in RIP-qPCR and MeRIP-qPCR.

**Additional file 3:** Raw blot images.

## Data Availability

The datasets supporting the conclusions of this article are available from the corresponding author on reasonable request.

## Abbreviations

ALKBH5: AlkB homolog 5
AP-1: Tivator protein-1
CAMKII: Calmodulin dependent protein kinase II
cAMP: Cyclic adenosine monophosphate
CREB: CAMP responsive element binding protein
FOXL2: Forkhead box l2
FOXP2: Forkhead box p2
FTO: Fat mass and obesity-associated protein
FSH: Follicle-stimulating hormone
FSHR: Follicle-stimulating hormone receptor
GnRH: Gonadotropin-releasing hormone
GnRHR: Gonadotropin-releasing hormone receptor
GPCR: G protein-coupled receptors
HPGA: Hypothalamic–pituitary–gonadal axis
LH: Luteinizing hormone
LHCGR: Luteinizing hormone/choriogonadotropin receptor
MAPK: Mitogen-activated protein kinase
METTL3: Methyltransferase-like 3
METTL14: Methyltransferase-like 14
m^6^A: N6-methyladenosine
PKA: Protein kinase A
PKC: Protein kinase C
YTHDF1: YTH Domain Family Protein 1
YTHDF2: YTH Domain Family Protein 2
YTHDF3: YTH Domain Family Protein 3
