# Supplementary material for: GnRH-driven FTO-mediated RNA m6A modification promotes gonadotropin synthesis and secretion
Source: BMC Biol. 2024 May 3;22:104. doi: 10.1186/s12915-024-01905-1 (PMC11069278; doi:10.1186/s12915-024-01905-1)
Supplement: Supplementary file 1 — Additional file 1: Fig. S1. GnRH promotes rat gonadotropin synthesis and secretion. Fig. S2. GnRH promotes gonadotropin synthesis and secretion and FTO expression in LβT2 cells and primary rat adenohypophysis cells. Fig. S3. Expression distribution of Foxp2 mRNA in rat pituitary and the distribution of its upper m6A modifications. Fig. S4. Validation of FOXP2 interactions with Fshb DNA. Fig. S5. Apoptosis of LβT2 cells and primary rat adenohypophysis cells after FOXP2 knockdown or overexpress. Fig. S6. FOXP2 regulates gonadotropin synthesis and secretion via activating the cAMP/PKA signaling pathway in primary rat adenohypophysis cells. Fig. S7. GnRH stimulation is dependent on FTO expression. Fig. S8. Decreased gonadotropin synthesis and secretion in Fto+/− mice. Fig. S9. Transfection efficiency assay of siRNAs and plasmids by RT-qPCR. [file 12915_2024_1905_MOESM1_ESM.docx]

**Additional file 1**

**GnRH-driven FTO-mediated RNA m^6^A modification promotes gonadotropin synthesis and secretion**

**Hao-Qi Wang, Yi-Ran Ma, Yu-Xin Zhang, Fan-Hao Wei, Yi Zheng, Zhong-Hao Ji, Hai-Xiang Guo, Tian Wang, Jia-Bao Zhang*, Bao Yuan***

**
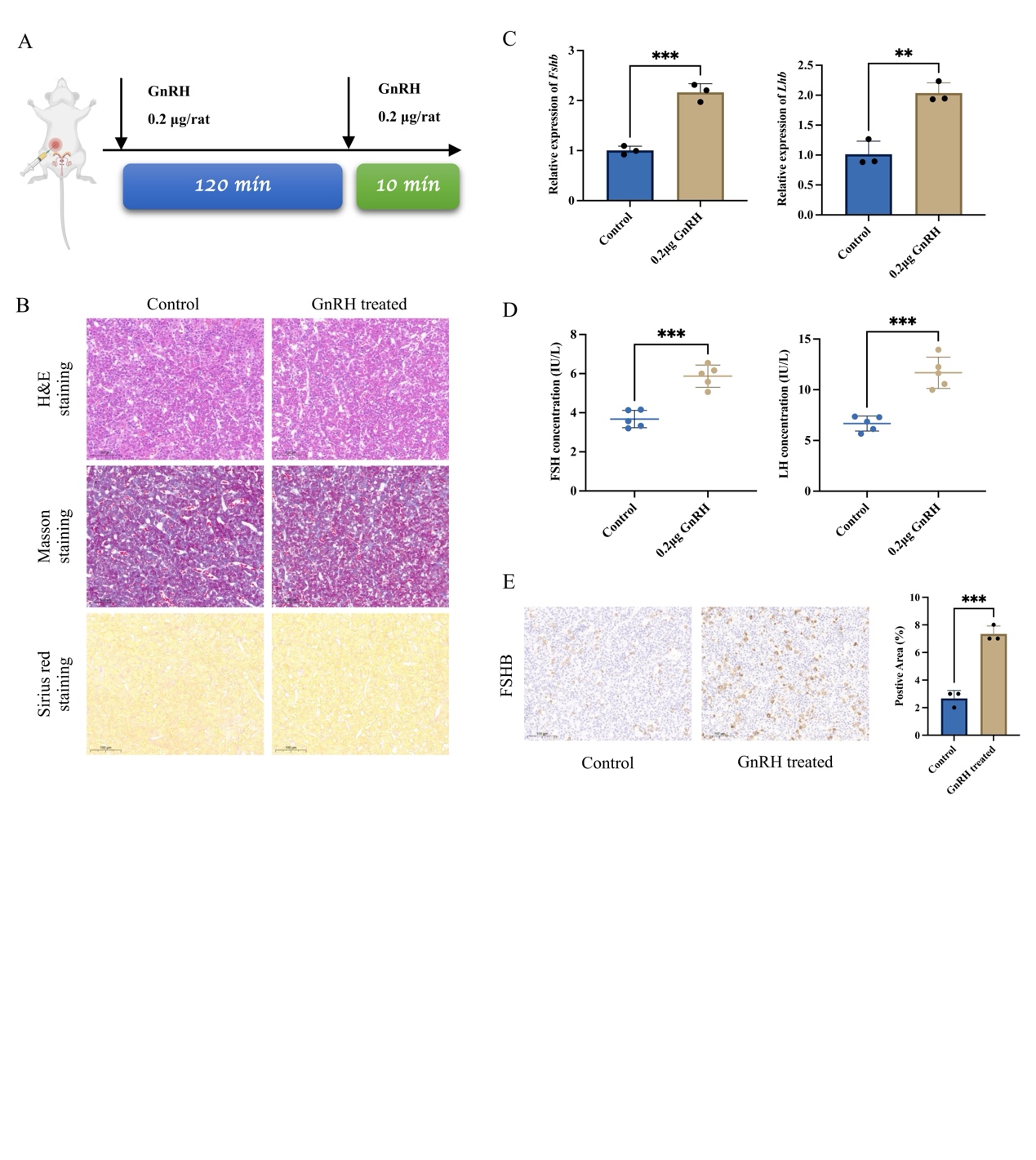
**

**Fig. S1.** **GnRH promotes rat gonadotropin synthesis and secretion. (A)** Program pattern diagram of twice 0.2 μg GnRH treatment. **(B)** Tissue section staining of rat adenohypophysis after GnRH treatment. **(C)** RT-qPCR to detect the expression of *Fshb* and *Lhb* mRNA in rat adenohypophysis after 0.2 μg GnRH treatment (n=3). **(D)** ELISA to detect the secretion of FSH and LH in rats after 0.2 μg GnRH treatment (n=5). **(E)** Immunohistochemistry to detect the expression of FSHB protein in rat adenohypophysis after 0.2 μg GnRH treatment (n=3). Statistical analysis was performed using unpaired t test; mean ± SD; **, *P*<0.01; ***, *P*<0.001.

**
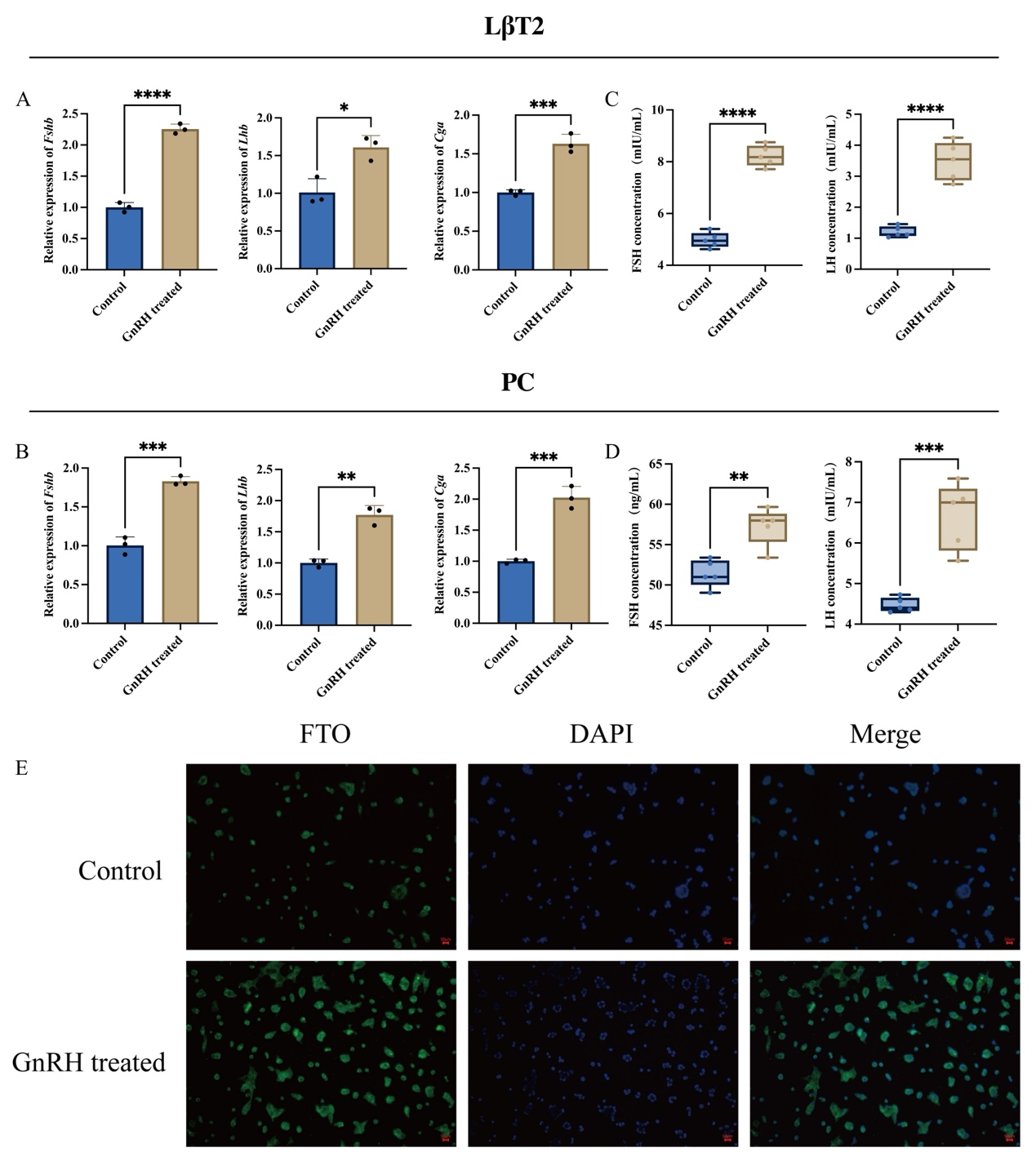
**

**Fig. S2.** **GnRH promotes gonadotropin synthesis and secretion and FTO expression in LβT2 cells and primary rat adenohypophysis cells. (A, B)** RT-qPCR analysis *Fshb, Lhb, and Cga* mRNA expression after GnRH treatment in LβT2 cells (A, n=3) and primary rat adenohypophysis cells (B, n=3). **(C, D)** ELISA analysis secretion levels of FSH and LH after GnRH treatment in LβT2 cells (C, n=5) and primary rat adenohypophysis cells (D, n=5). **(E)** Immunofluorescence analysis FTO after GnRH treatment in LβT2 cells. Statistical analysis was performed using unpaired t test; mean ± SD; **, *P*<0.01; ***, *P*<0.001.

**
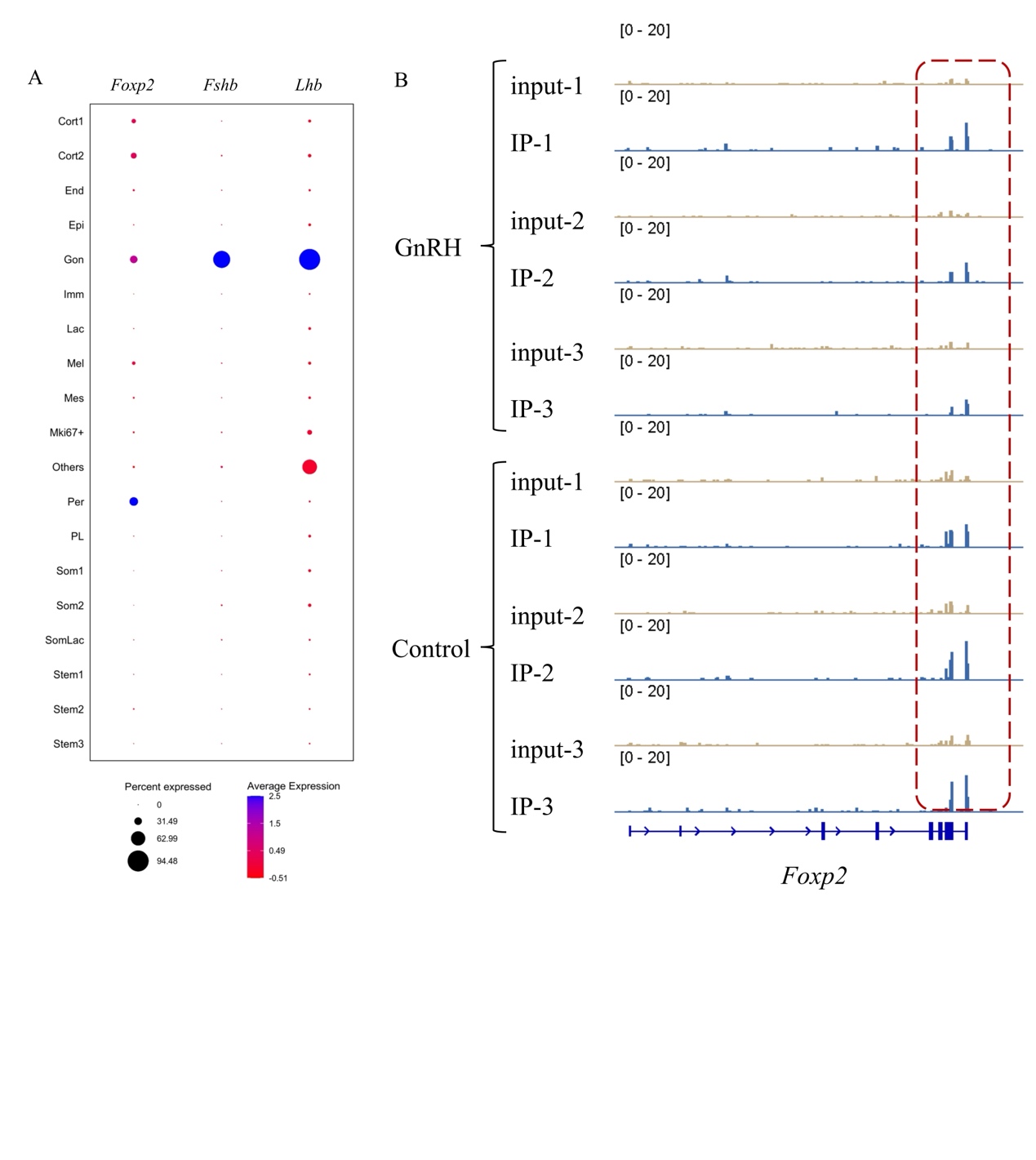
**

**Fig. S3.** **Expression distribution of *Foxp2* mRNA in rat pituitary and the distribution of its upper m^6^A modifications. (A)** Pituitary single-cell transcriptomic analysis of *Foxp2*, *Fshb,* and *Lhb* mRNA enrichment in different cell populations. **(B)** IGV peak maps of m^6^A modifications on adenohypophyseal *Foxp2* mRNA after GnRH treatment based on m^6^A-seq. Cort, corticotropes; End, endothelial; Epi, epithelial; Gon, gonadotrope; Imm, immune cells; Lac, lactotropes; Mel, melanotropes; Mes, mesenchymal; Mki67+, Mki67^+^ proliferating epithelial cells; Per, pericytes; PL, posterior lobe; Som, somatotropes; SomLac, somatolactotropes; Stem, stem cells; Others, other cells.

**
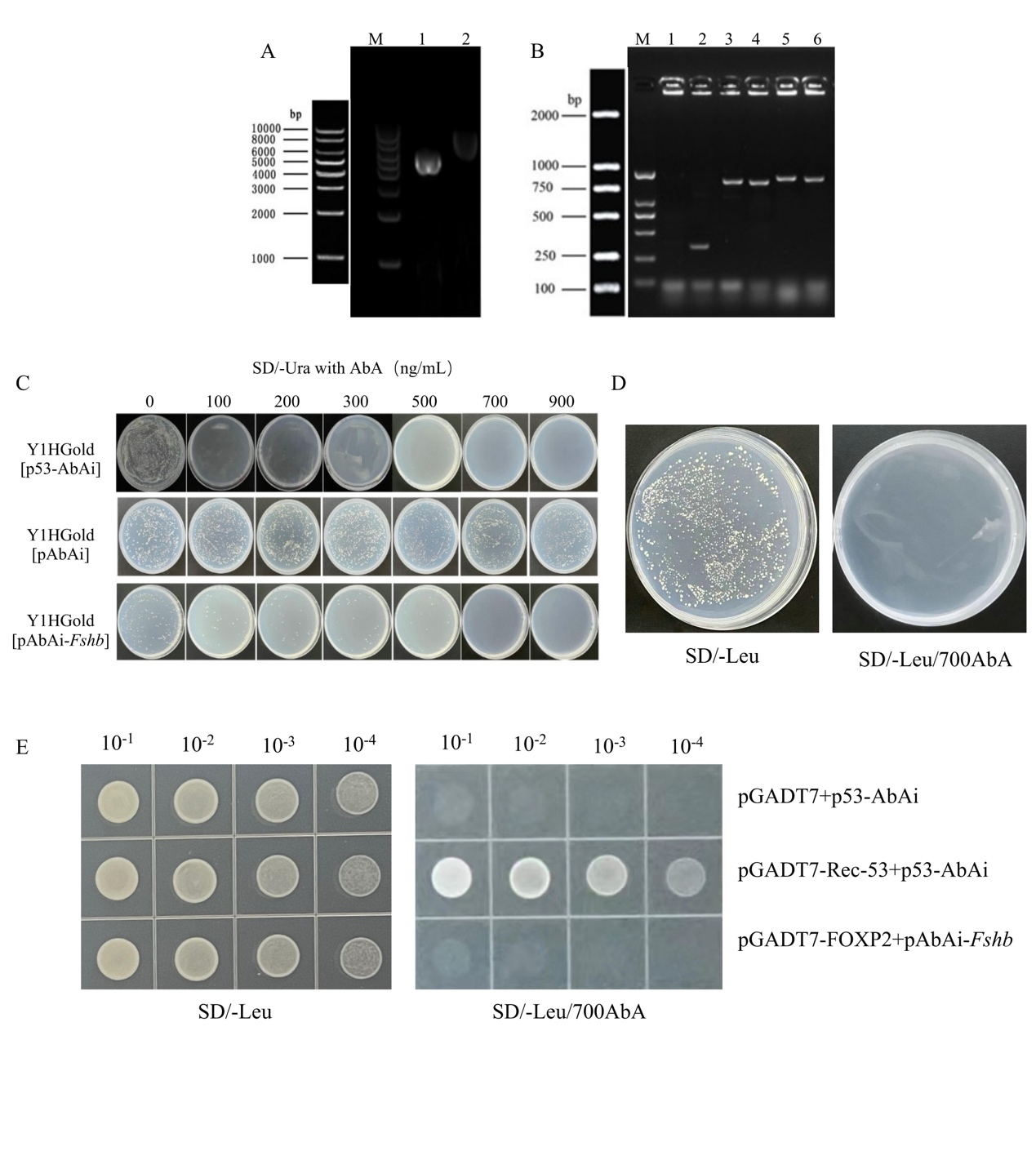
**

**Fig. S4.** **Validation of FOXP2 interactions with *Fshb* DNA.** **(A)** Linearization assay of the recombinant plasmid pAbAi-*Fshb*. LineM, Marker; Line1, pre-enzyme plasmid; Line2, post-enzyme plasmid. **(B)** Integration identification of the decoy strain Y1HGold[pAbAi-*Fshb*]. LineM, Marker; Line1, ddH_2_O; Line2, Y1HGold[pAbAi]; Line3 to 5, Y1HGold[pAbAi- *Fshb*]. **(C)** Self-activation detection of Y1HGold[pAbAi-*Fshb*]. **(D)** Point-to-point verification of FOXP2 and *Fshb* interoperation. **(E)** Point plate verification of FOXP2 and *Fshb* interoperation.

**
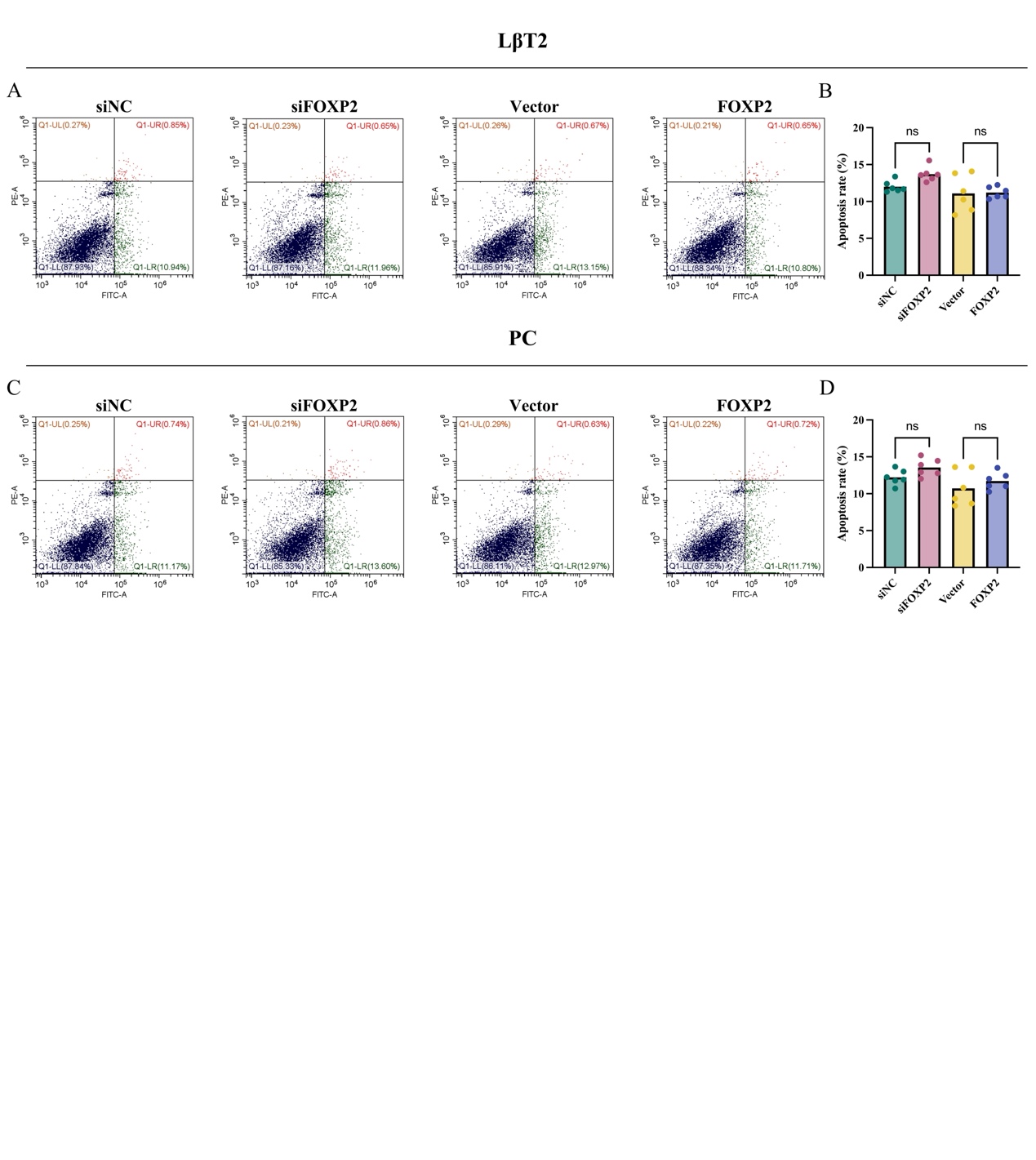
**

**Fig. S5.** **Apoptosis of LβT2 cells and primary rat adenohypophysis cells after FOXP2 knockdown or overexpress. (A)** Apoptosis of LβT2 cells after FOXP2 knockdown or overexpress. **(B)** Statistical analysis of apoptosis of LβT2 cells (n=6). **(C)** Apoptosis of primary rat adenohypophysis cells after FOXP2 knockdown or overexpress. **(D)** Statistical analysis of apoptosis of primary rat adenohypophysis cells (n=6). "ns" represents no statistical difference.

**
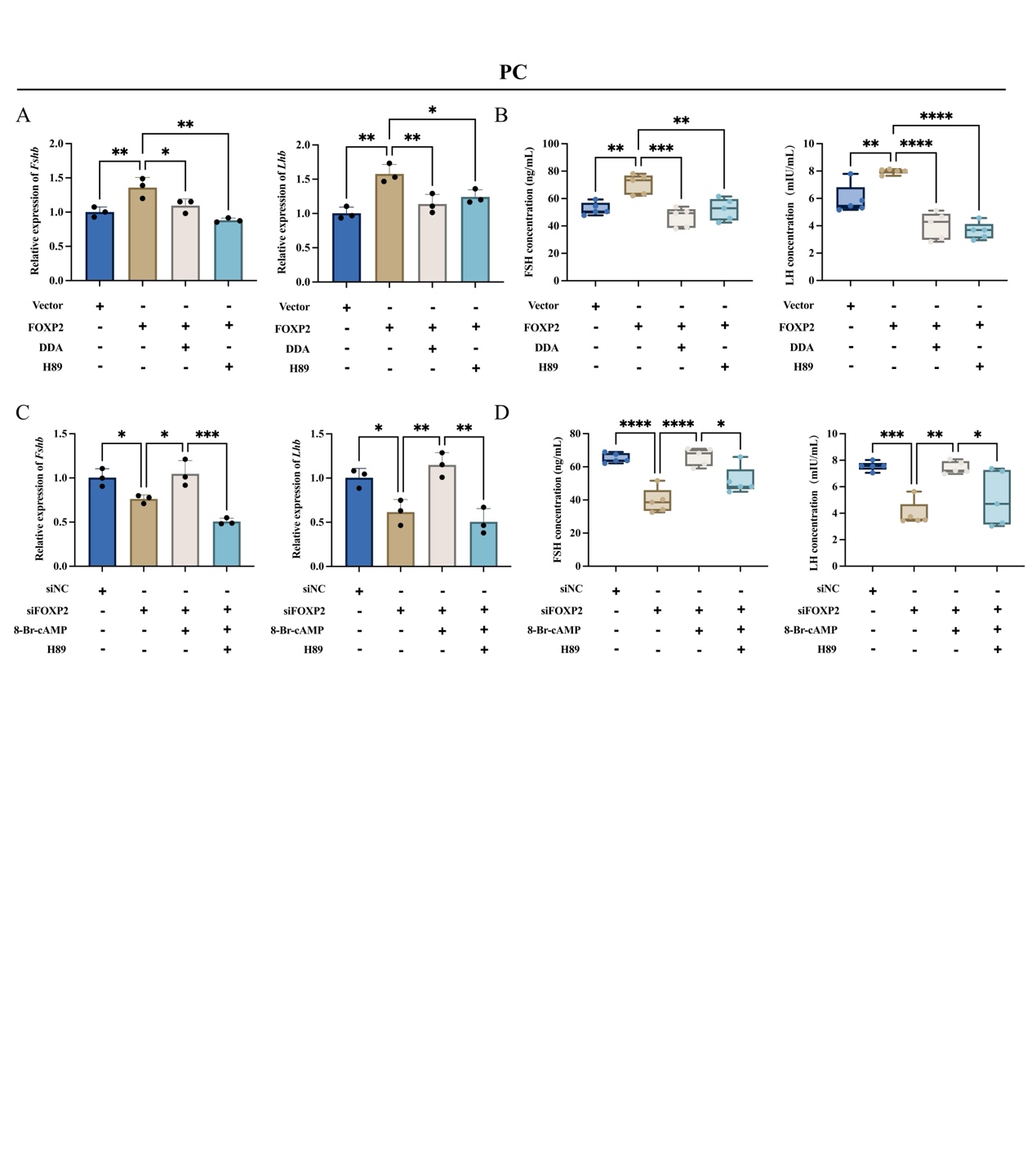
**

**Fig. S6.** **FOXP2 regulates gonadotropin synthesis and secretion via activating the cAMP/PKA signaling pathway in** **primary rat adenohypophysis cells.** **(A, B)** FOXP2 plasmid or vector was transfected into primary rat adenohypophysis cells with or without the indicated compounds (DDA, 10 μM; H89, 10 μM). The expression of *Fshb* and *Lhb* mRNA was assayed by RT-qPCR (A, n=3). The secretion of FSH and LH was assayed by ELISA (B, n=5). **(C, D)** FOXP2 siRNA or siNC were transfected into primary rat adenohypophysis cells with or without the indicated compounds (8-Bromo-cAMP, 500 μM; H89, 10 μM). The expression of *Fshb* and *Lhb* mRNA was assayed by RT-qPCR (C, n=3). The secretion of FSH and LH was assayed by ELISA (D, n=5). Statistical analysis was performed using one-way ANOVA; mean ± SD; *, *P*<0.05; **, *P*<0.01; ***, *P*<0.001; ****, *P*<0.0001.

**
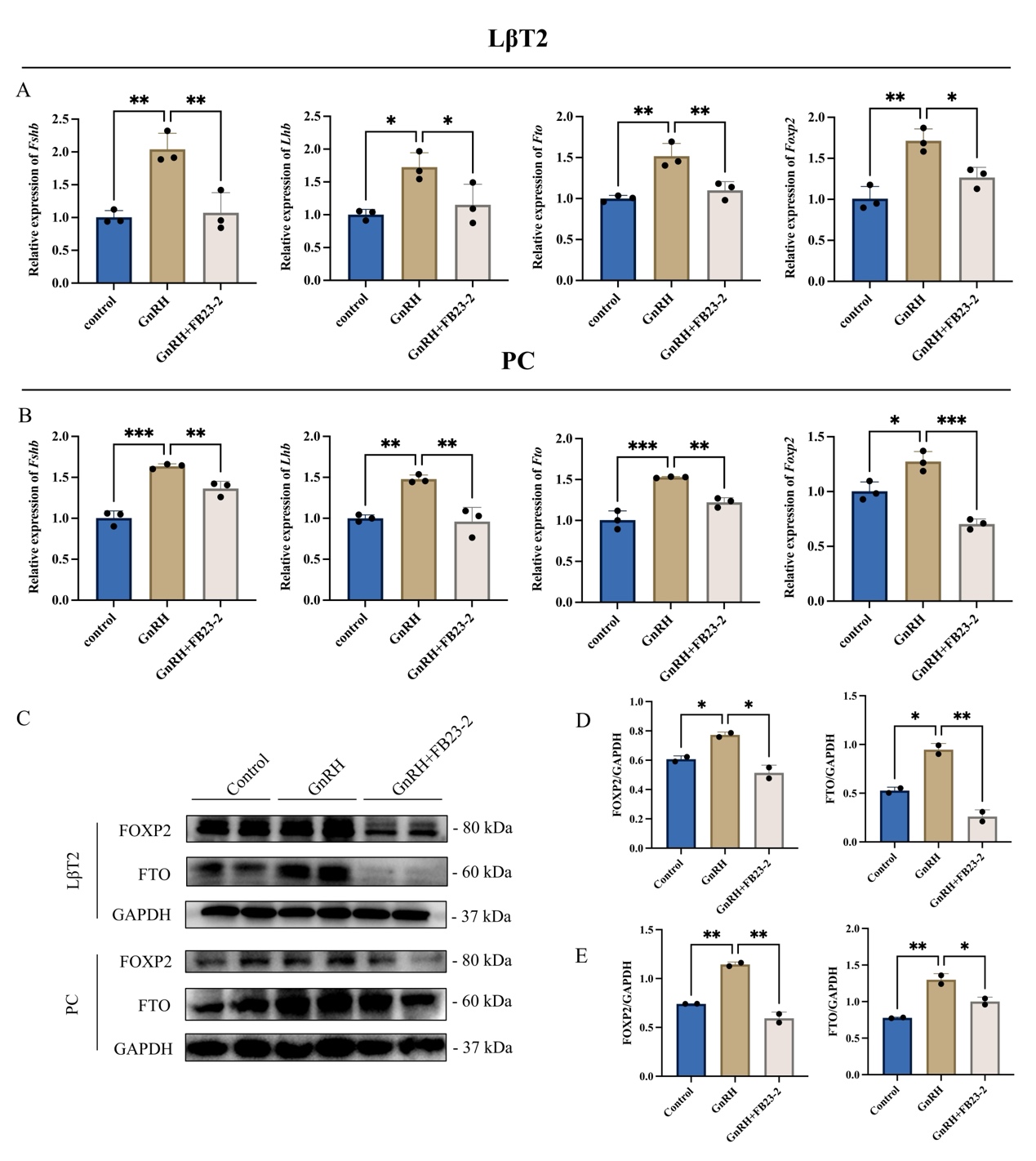
**

**Fig. S7.** **GnRH stimulation is dependent on FTO expression. (A, B)** RT-qPCR to detect the expression of *Fshb*, *Lhb*, *Fto,* and *Foxp2* mRNA in LβT2 cells (A, n=3) and primary rat adenohypophysis cells (B, n=3). **(C-E)** WB analysis FTO and FOXP2 in LβT2 cells and primary rat adenohypophysis cells (C, n=2). Statistical analysis of FTO and FOXP2 protein expression changes in LβT2 cells (D) and primary rat adenohypophysis cells (E). Statistical analysis was performed using one-way ANOVA; mean ± SD; *, *P*<0.05; **, *P*<0.01; ***, *P*<0.001.

**
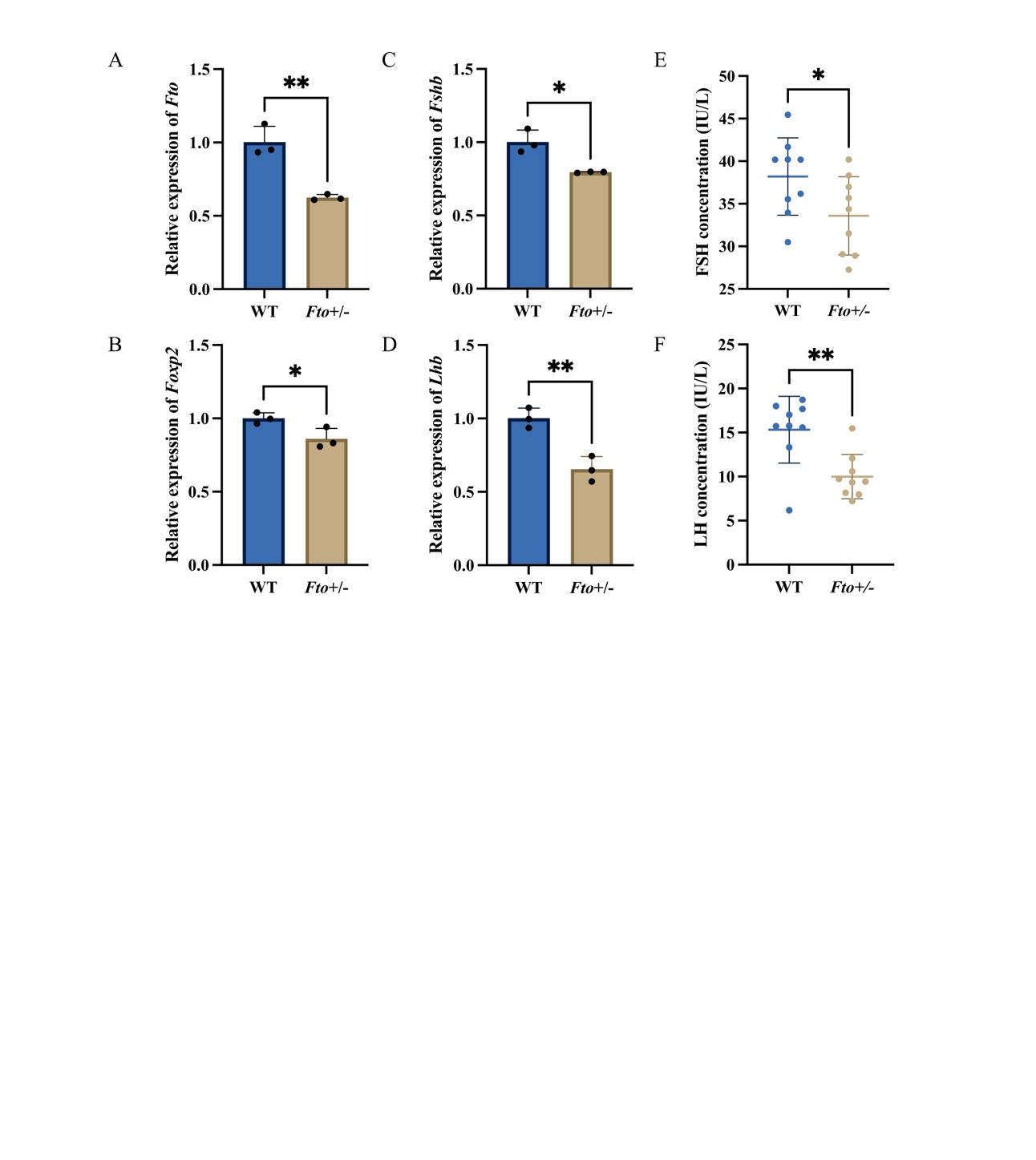
**

**Fig. S8.** **Decreased gonadotropin synthesis and secretion in** ***Fto*^+/−^ mice. (A-D)** RT-qPCR to detect the mRNA expression of *Fto* (A, n=3), *Foxp2* (B, n=3), *Fshb* (C, n=3), and *Lhb* (D, n=3) in *Fto*^+/−^ mice pituitary tissues. **(E, F)** ELISA to detect the secretion of FSH (E, n=9) and LH (F, n=9) in *Fto*^+/−^ mice. Statistical analysis was performed using unpaired t test; mean ± SD; *, *P*<0.05; **, *P*<0.01.

**
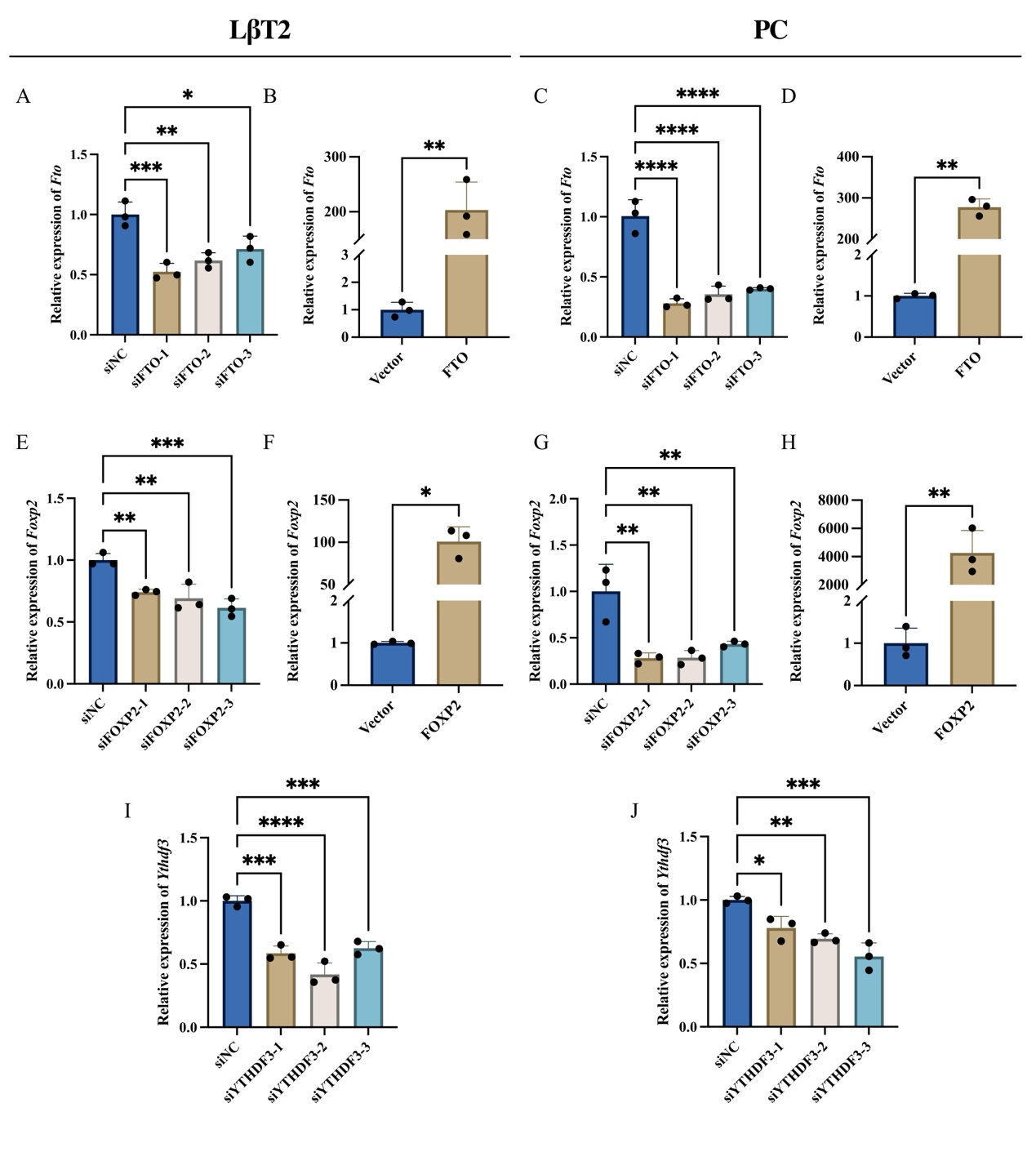
**

**Fig. S9.** **Transfection efficiency assay of siRNAs and plasmids by RT-qPCR. (A)** RT-qPCR analysis *Fto* mRNA expression in LβT2 cells transfected with siNC and three FTO siRNAs (n=3). **(B)** RT-qPCR analysis *Fto* mRNA expression in LβT2 cells transfected with vector and FTO plasmid (n=3). **(C)** RT-qPCR analysis *Fto* mRNA expression in primary rat adenohypophysis cells transfected with siNC and three FTO siRNAs (n=3). **(D)** RT-qPCR analysis *Fto* mRNA expression in primary rat adenohypophysis cells transfected with vector and FTO plasmid (n=3). **(E)** RT-qPCR analysis *Foxp2* mRNA expression in LβT2 cells transfected with siNC and three FOXP2 siRNAs (n=3). **(F)** RT-qPCR analysis *Foxp2* mRNA expression in LβT2 cells transfected with vector and FOXP2 plasmid (n=3). **(G)** RT-qPCR analysis *Foxp2* mRNA expression in primary rat adenohypophysis cells transfected with siNC and three FOXP2 siRNAs (n=3). **(H)** RT-qPCR analysis *Foxp2* mRNA expression in primary rat adenohypophysis cells transfected with vector and FOXP2 plasmid (n=3). (I) RT-qPCR analysis *Ythdf3* mRNA expression in LβT2 cells transfected with siNC and three YTHDF3 siRNAs (n=3). (J) RT-qPCR analysis *Ythdf3* mRNA expression in primary rat adenohypophysis cells transfected with siNC and three YTHDF3 siRNAs (n=3). Statistical analysis was performed using unpaired t test; mean ± SD; *, *P*<0.05; **, *P*<0.01; ***, *P*<0.001; ****, *P*<0.0001.
