# Supplementary material for: GnRH-driven FTO-mediated RNA m6A modification promotes gonadotropin synthesis and secretion
Source: BMC Biol. 2024 May 3;22:104. doi: 10.1186/s12915-024-01905-1 (PMC11069278; doi:10.1186/s12915-024-01905-1)
Supplement: Supplementary file 2 — Additional file 2: Table S1. Sequences of siRNAs used in the study. Table S2. Mouse primers used in the study. Table S3. Rat primers used in the study. Table S4. Enzyme-cutting system for plasmid linearization. Table S5. Primers used in RIP-qPCR and MeRIP-qPCR. [file 12915_2024_1905_MOESM2_ESM.docx]

**Additional file 2**

**GnRH-driven FTO-mediated RNA m^6^A modification promotes gonadotropin synthesis and secretion**

**Hao-Qi Wang, Yi-Ran Ma, Yu-Xin Zhang, Fan-Hao Wei, Yi Zheng, Zhong-Hao Ji, Hai-Xiang Guo, Tian Wang, Jia-Bao Zhang*, Bao Yuan***

**Table S1.** **Sequences of siRNAs used in the study.**

| **Name** | **siRNA sequence (5'-3')** |
| --- | --- |
| si-NC | UUCUUCGAACGUGUCACGUTT |
| Mus-si-FTO-1 | GCAGCUGAAAUACCCUAAATT |
| Mus-si-FTO-2 | GCACCUACAAGUACUUGAATT |
| Mus-si-FTO-3 | CAGGCACCUUGGAUUAUAUTT |
| Mus-si-FOXP2-1 | GCAAACCAGUGGAUUGAAATT |
| Mus-si-FOXP2-2 | GCUGGUUUACACGGACAUUTT |
| Mus-si-FOXP2-3 | GCUGAGAUUCAGCAACUAUTT |
| Mus-si-YTHDF3-1 | CCAAUGUCAGAUCCAUAUATT |
| Mus-si-YTHDF3-2 | GCAGUGGUAUGACUAGCAUTT |
| Mus-si-YTHDF3-3 | CCUCAAUCUUUGAUGACUUTT |
| Rat-si-FTO-1 | GCUGAAAUAUCCUAAACUGTT |
| Rat-si-FTO-2 | CAAGCUCAAUGACUACCUATT |
| Rat-si-FTO-3 | GGCUUGACAAUUCCUCUUCTT |
| Rat-si-FOXP2-1 | AGCAGCAACAACUACAAGATT |
| Rat-si-FOXP2-2 | CAAAGCUUCACCGCCAAUATT |
| Rat-si-FOXP2-3 | CGACAUUCAGACAAAUACATT |
| Rat-si-YTHDF3-1 | CCACCAAUGUCAGAUCCAUTT |
| Rat-si-YTHDF3-2 | GCACCUAAACCAACUUCUUTT |
| Rat-si-YTHDF3-3 | CCUCAAUCUUUGAUGACUUTT |

**Table S2.** **Mouse primers used in the study.**

| **Gene** | **Forward Primer (5'-3')** | **Reverse Primer (5'-3')** |
| --- | --- | --- |
| *Cga* | CATCACATGGAACCGGGACA | CAGTGGCACTCCGTATGATTCTC |
| *Foxp2* | GCTTTTCATGTGCTGTGCCA | CATCACATGGAACCGGGACA |
| *Fshb* | ACTGCTACACTAGGGATCTGG | CAGCCAGGCAATCTTACGGT |
| *Fto* | TCACAGCCTCGGTTTAGTTC | GCAGGATCAAAGGATTTCAACG |
| *Gapdh* | TGACGTGCCGCCTGGAGAAAC | CCGGCATCGAAGGTGGAAGAG |
| *Lhb* | AGGACTCAACCAATGGCCTG | GGAGGGAGGGAGGGATGATT |
| *Ythdf3* | GGGCAAGGAAATAAAGTTTCAGT | ACCATGCTGCTTCCCCAAG |

**Table S3. Rat primers used in the study.**

| **Gene** | **Forward Primer (5'-3')** | **Reverse Primer (5'-3')** |
| --- | --- | --- |
| *Cga* | GCCCAGAACACATCCTTCCA | TATGCAGGACCATGGACAGC |
| *Foxp2* | AGTGCCCAATGTGGGAG | CATGATAGCCTGCCTTATGAGTG |
| *Fshb* | ATACCACTTGGTGTGAGGGC | TAGAGGGAGTCTGAGTGGCG |
| *Fto* | TCTTACAACGCTGCCAGTTG | ACACCACCAATCAGTGCAAA |
| *Gapdh* | GGAAACCCATCACCATCTTC | GTGGTTCACACCCATCACAA |
| *Lhb* | CAAAAGCCAGGTCAGGGATA | GTACTCGAACCATGCTAGGACA |
| *Ythdf3* | CCTCACCAAGTGCAGTCTCA | CAGCACTGGATGCACCTCTA |

**Table S4.** **Enzyme-cutting system for** **plasmid linearization.**

| **Reaction component** | **Reaction system** |
| --- | --- |
| pAbAi-*Fshb* plasmid | 4 µg |
| 10×Buffer | 5 µL |
| BstBI | 1 µL |
| ddH_2_O | Up to 50 µL |

**Table S5.** **Primers used in RIP-qPCR and MeRIP-qPCR.**

| **Gene** | **Forward Primer (5'-3')** | **Reverse Primer (5'-3')** |
| --- | --- | --- |
| *Foxp2* | TGCTTTGGTCAGCCTTCTGT | AGGGCTAAATGAAAAGATCCCAGA |
