# Supplementary material for: GnRH-driven FTO-mediated RNA m6A modification promotes gonadotropin synthesis and secretion
Source: BMC Biol. 2024 May 3;22:104. doi: 10.1186/s12915-024-01905-1 (PMC11069278; doi:10.1186/s12915-024-01905-1)

Additional file 3

GnRH-driven FTO-mediated RNA m^{6}A modification promotes gonadotropin synthesis and secretion

Hao-Qi Wang, Yi-Ran Ma, Yu-Xin Zhang, Fan-Hao Wei, Yi Zheng, Zhong-Hao Ji, Hai-Xiang Guo, Tian Wang, Jia-Bao Zhang*, Bao Yuan*

Fig.1I

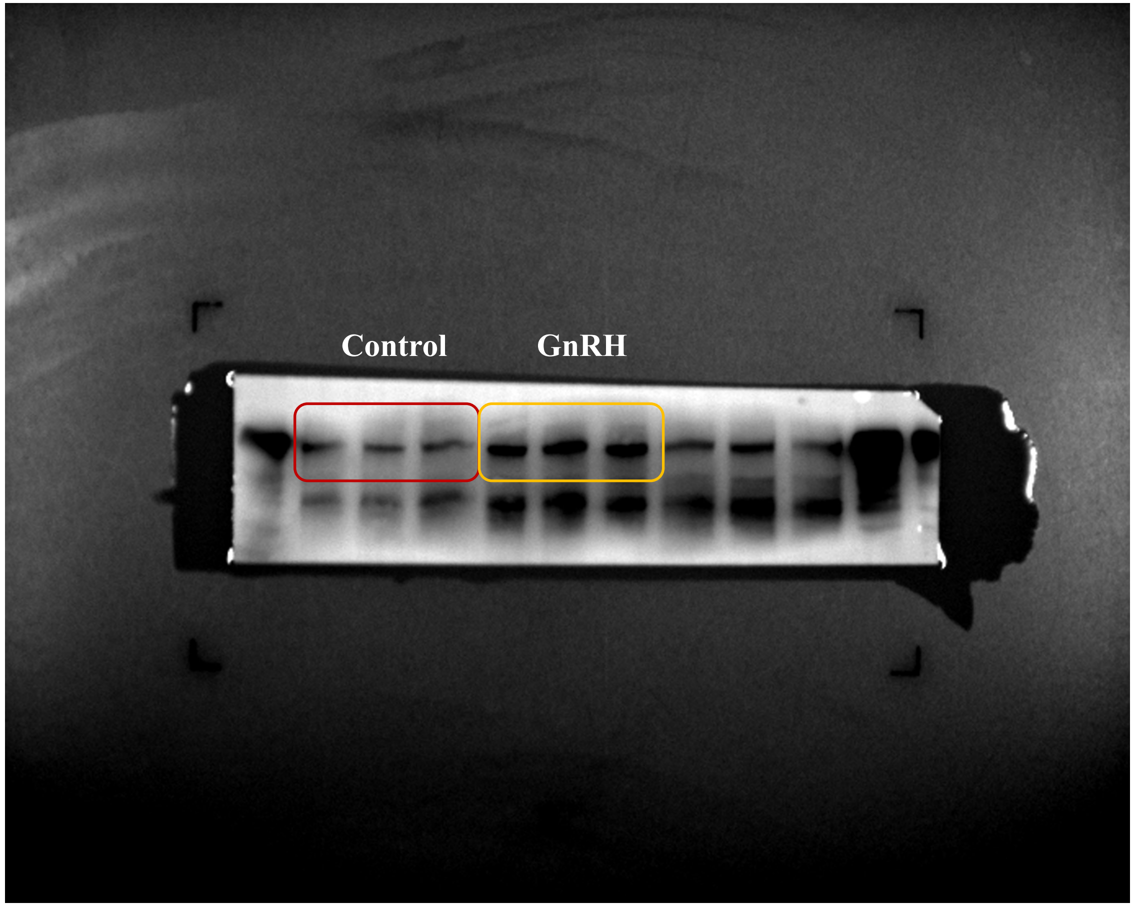

FTO

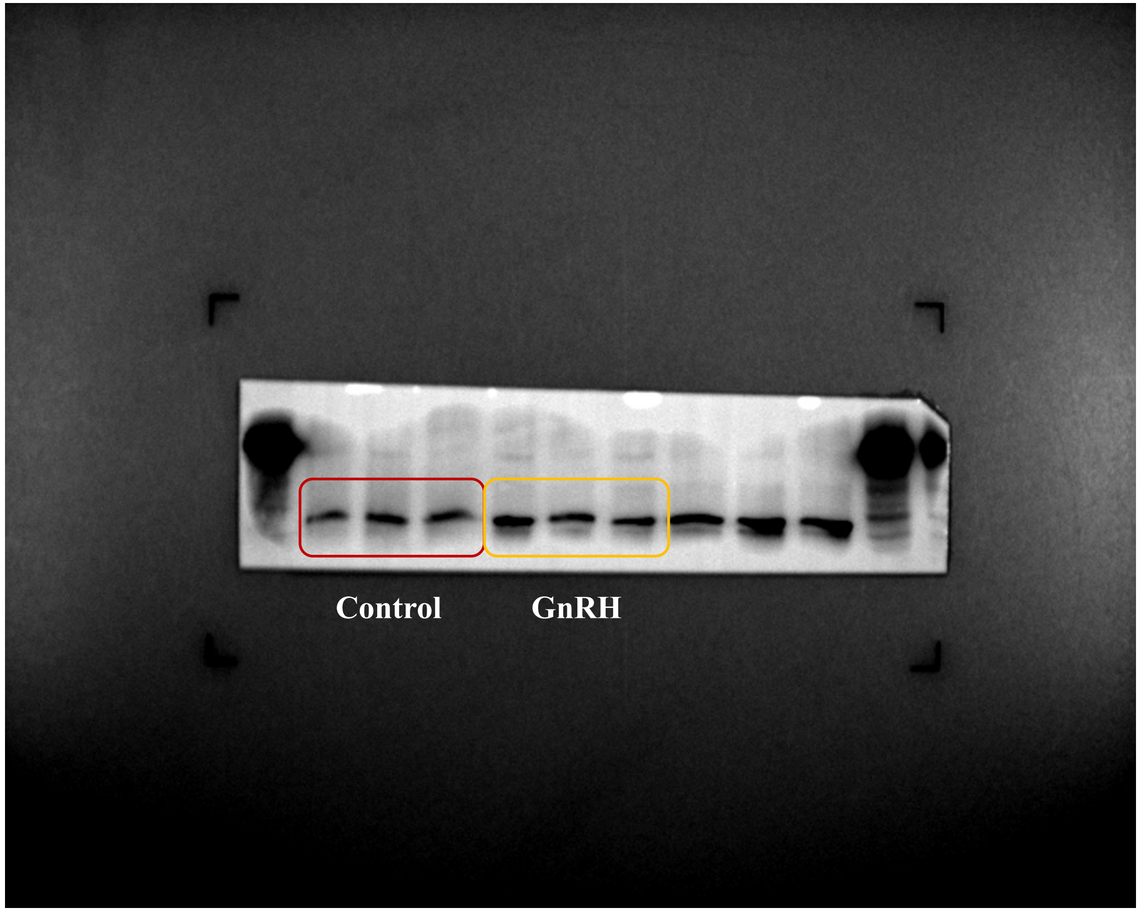

GAPDH

Fig.1J

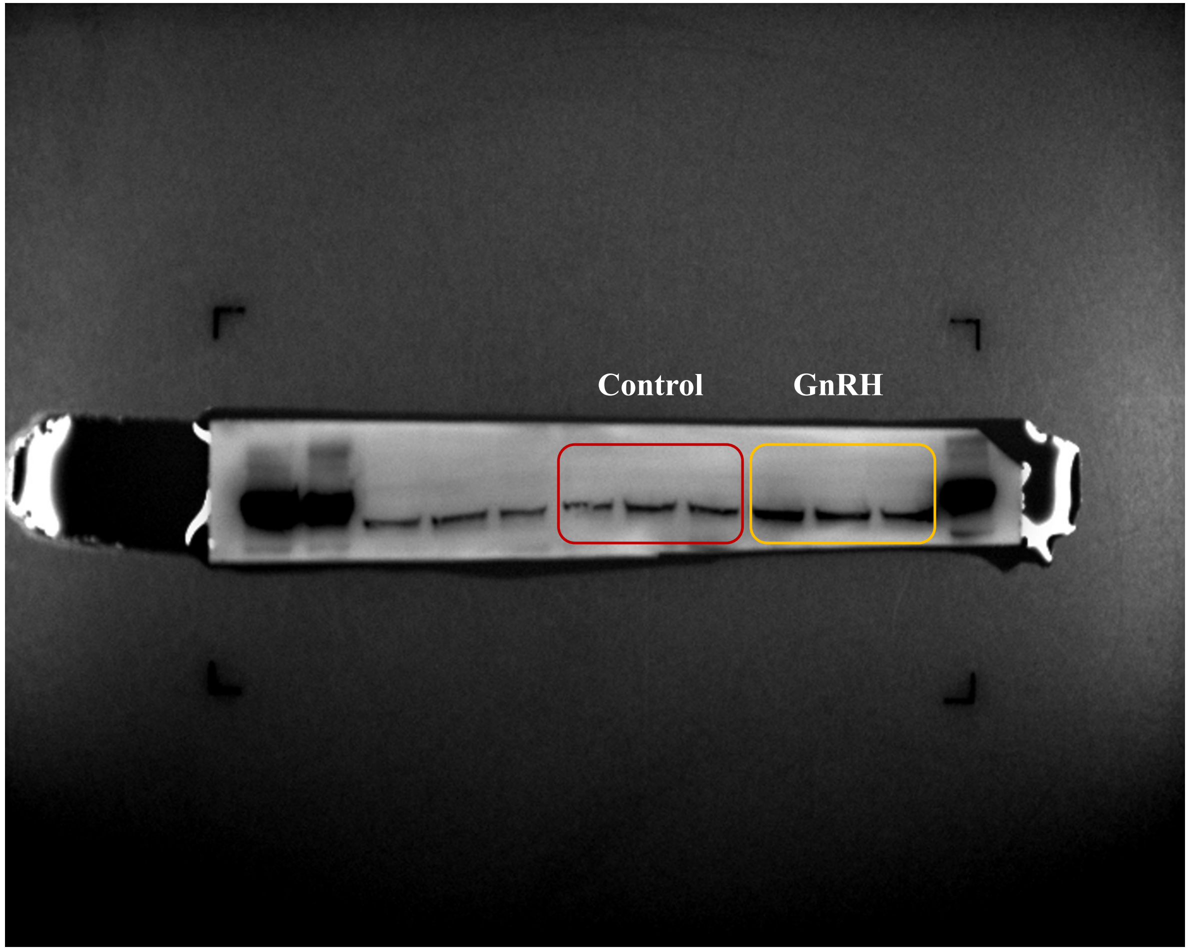

FTO

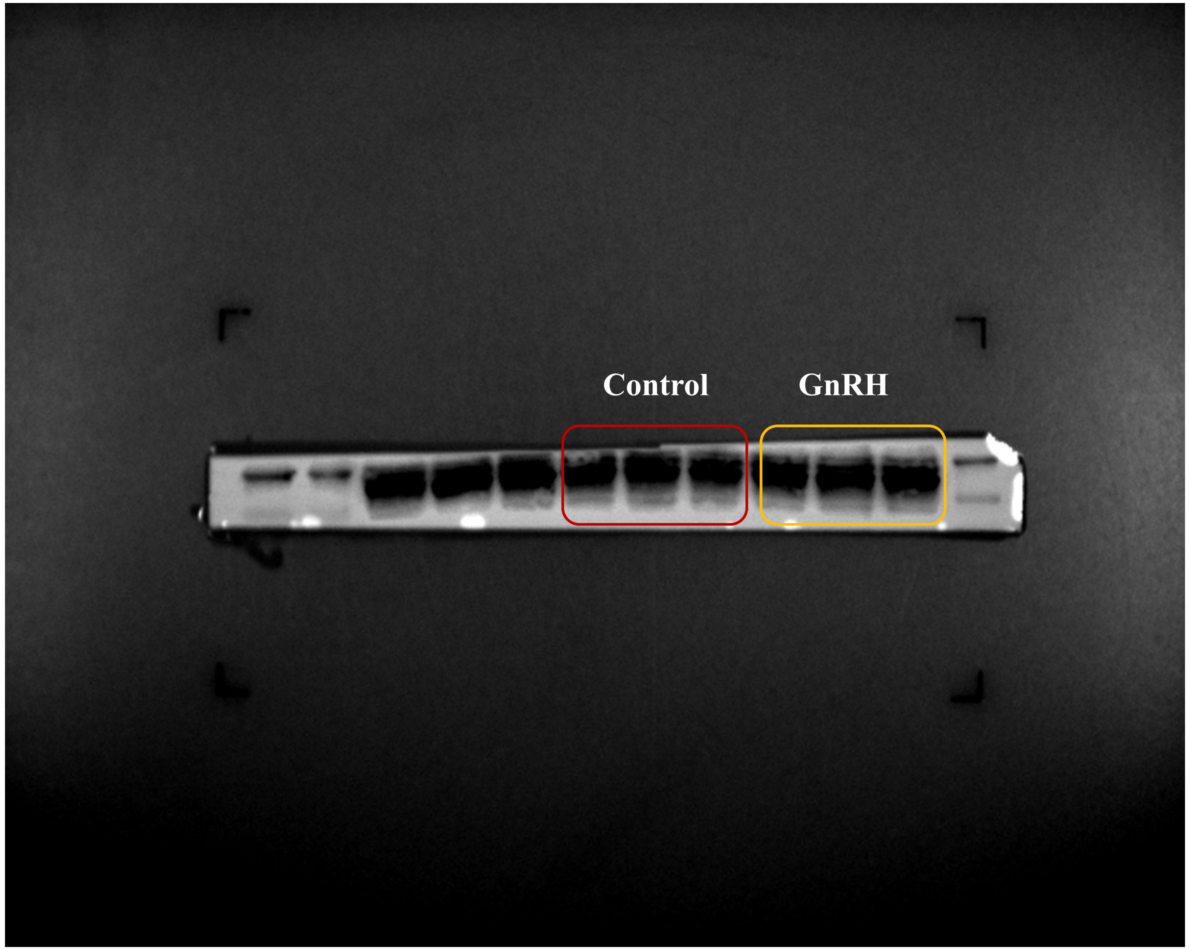

GAPDH

Fig.2G: LβT2

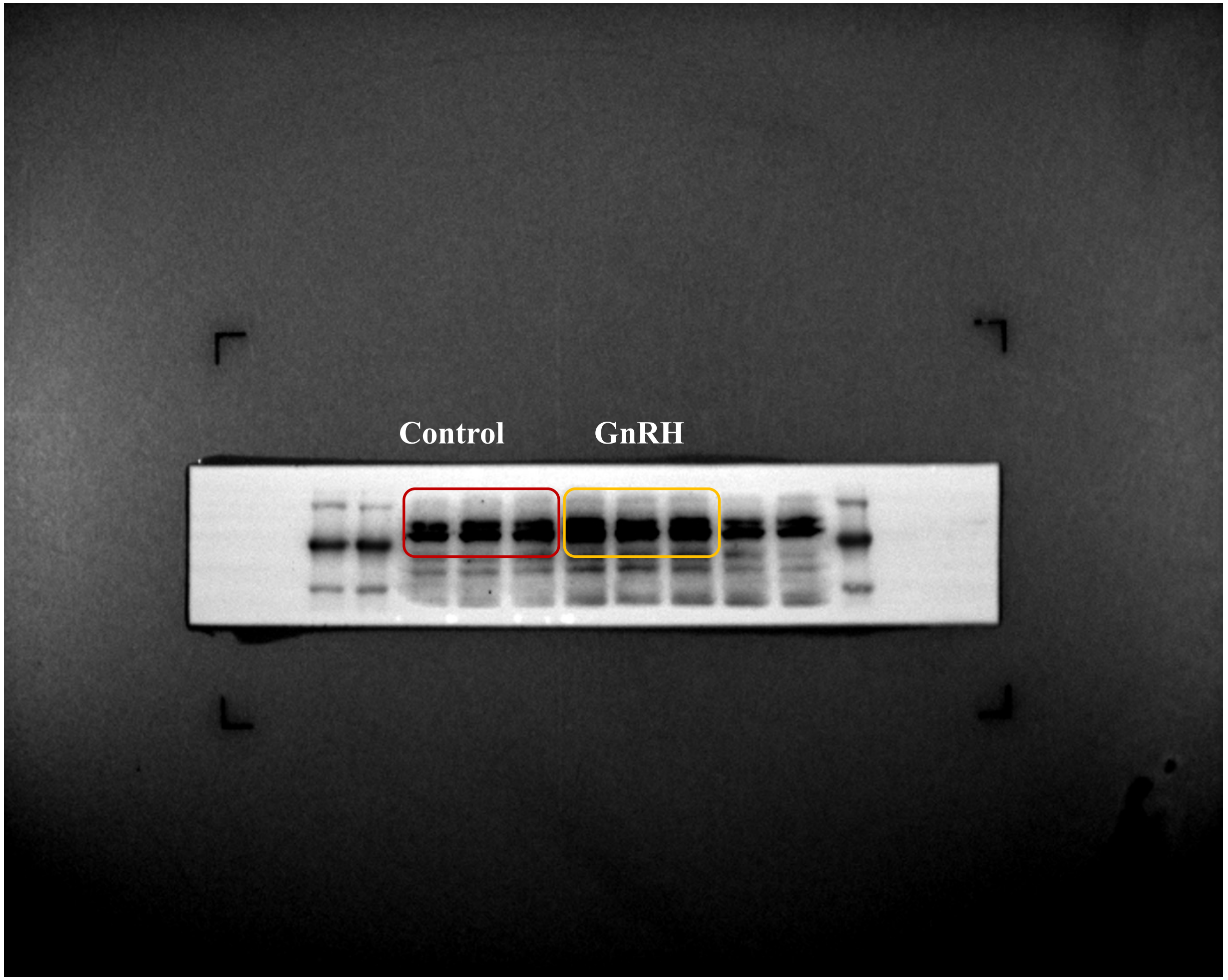

FOXP2

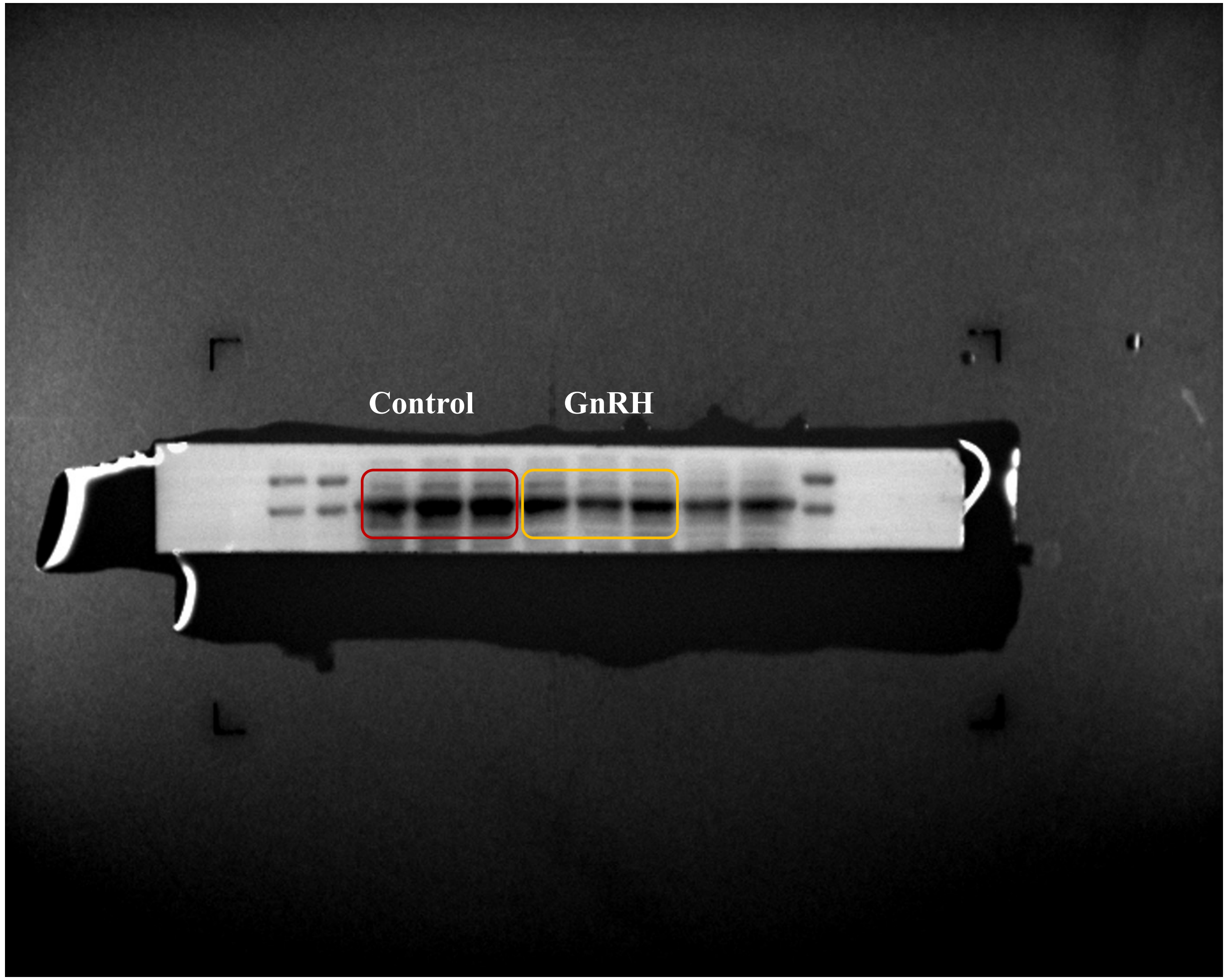

GAPDH

Fig.2G: PC

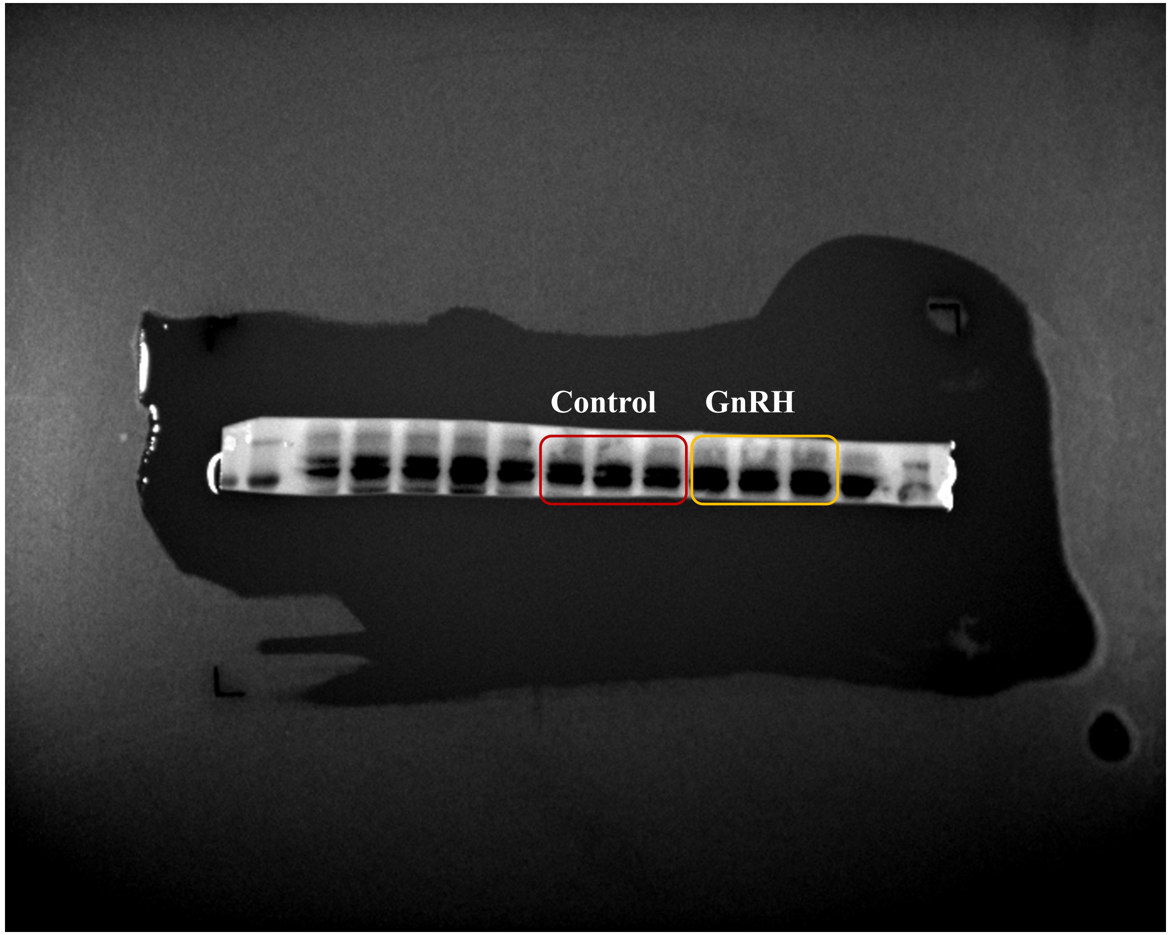

FOXP2

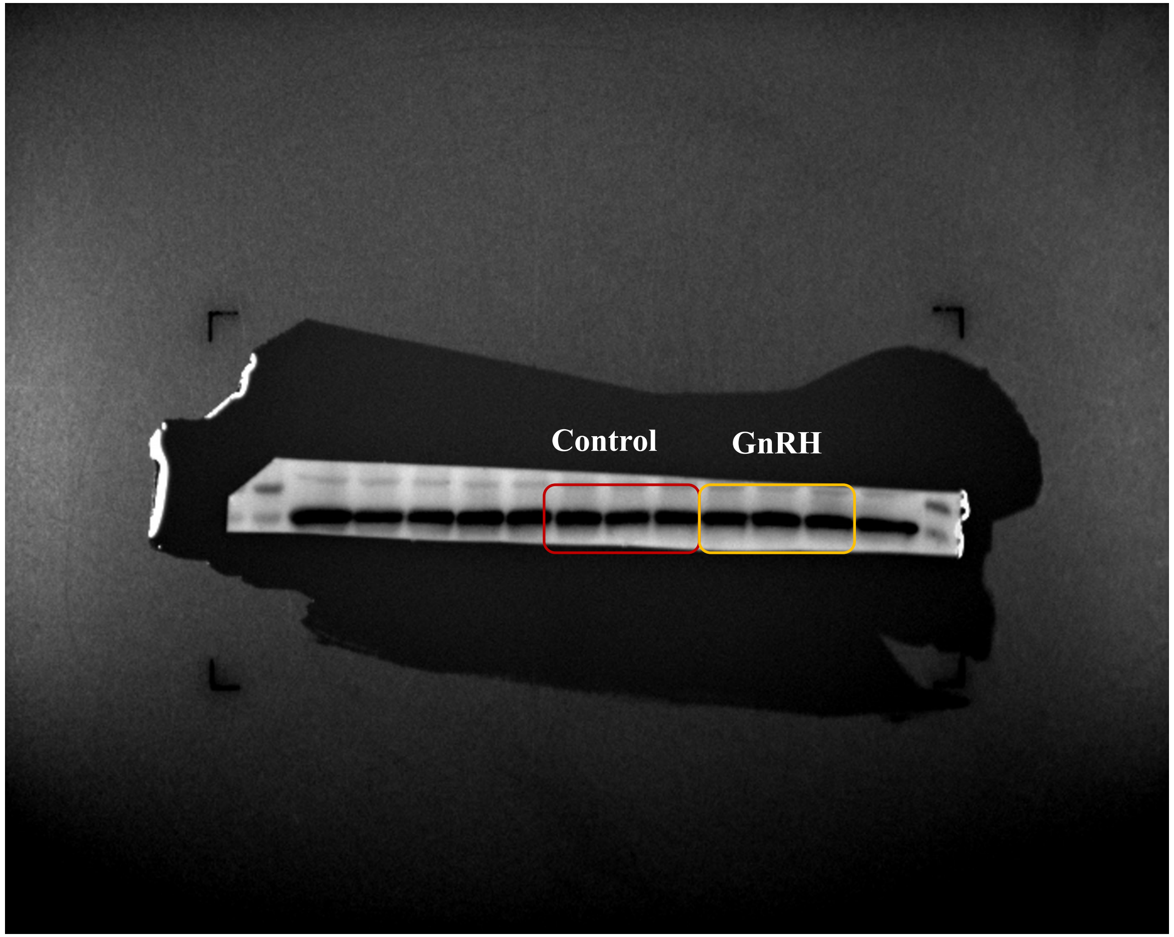

GAPDH

Fig.3A: LβT2

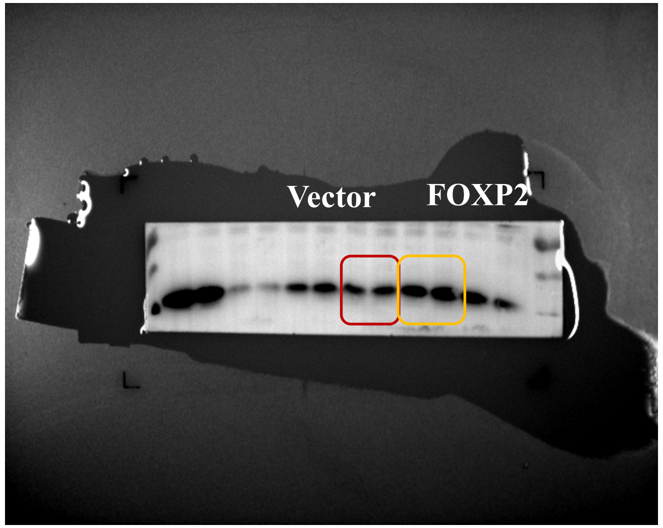

cAMP

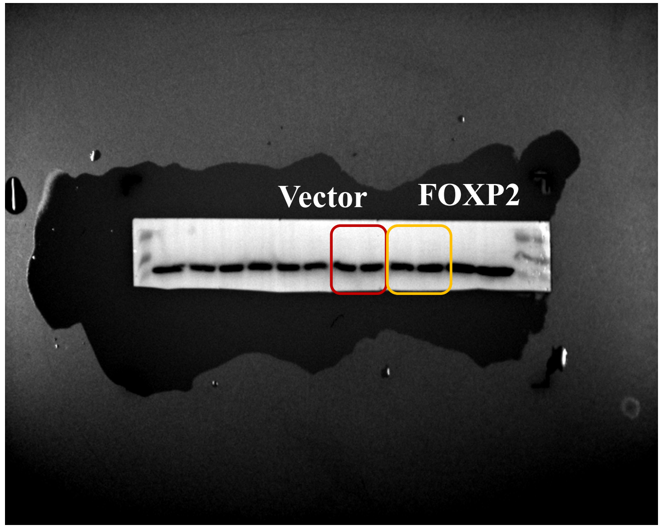

GAPDH

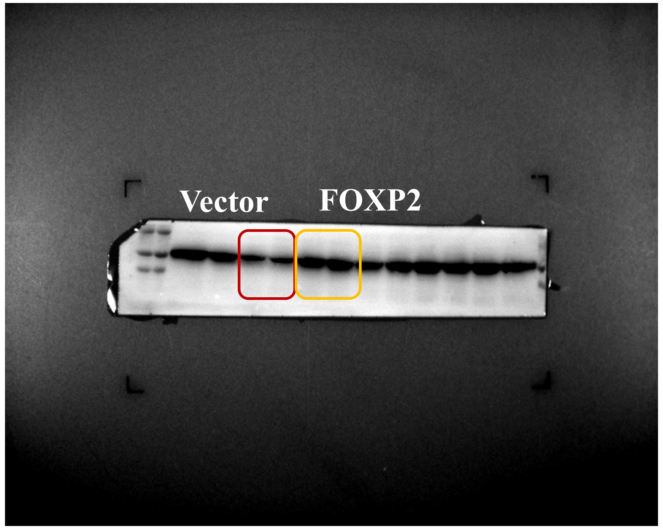

p-PKA

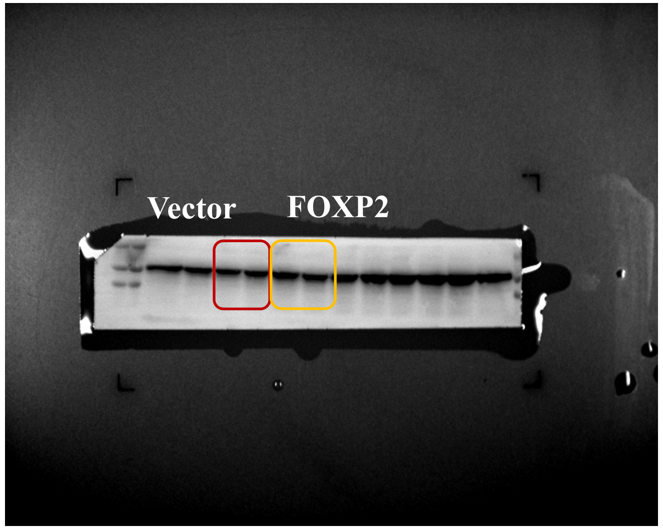

PKA

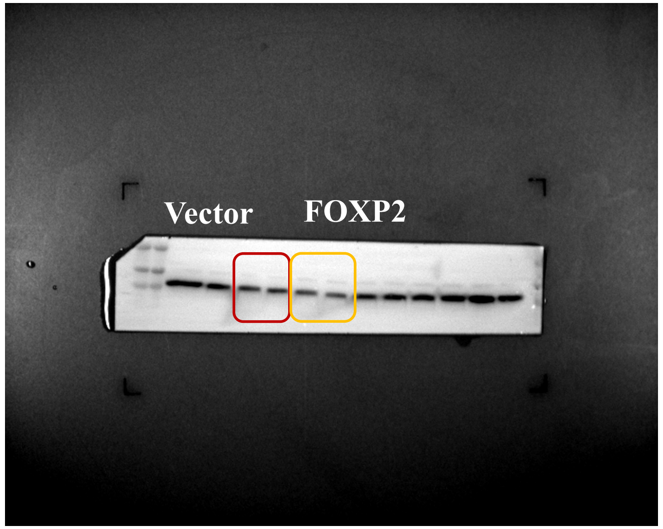

GAPDH

Fig.3A: PC

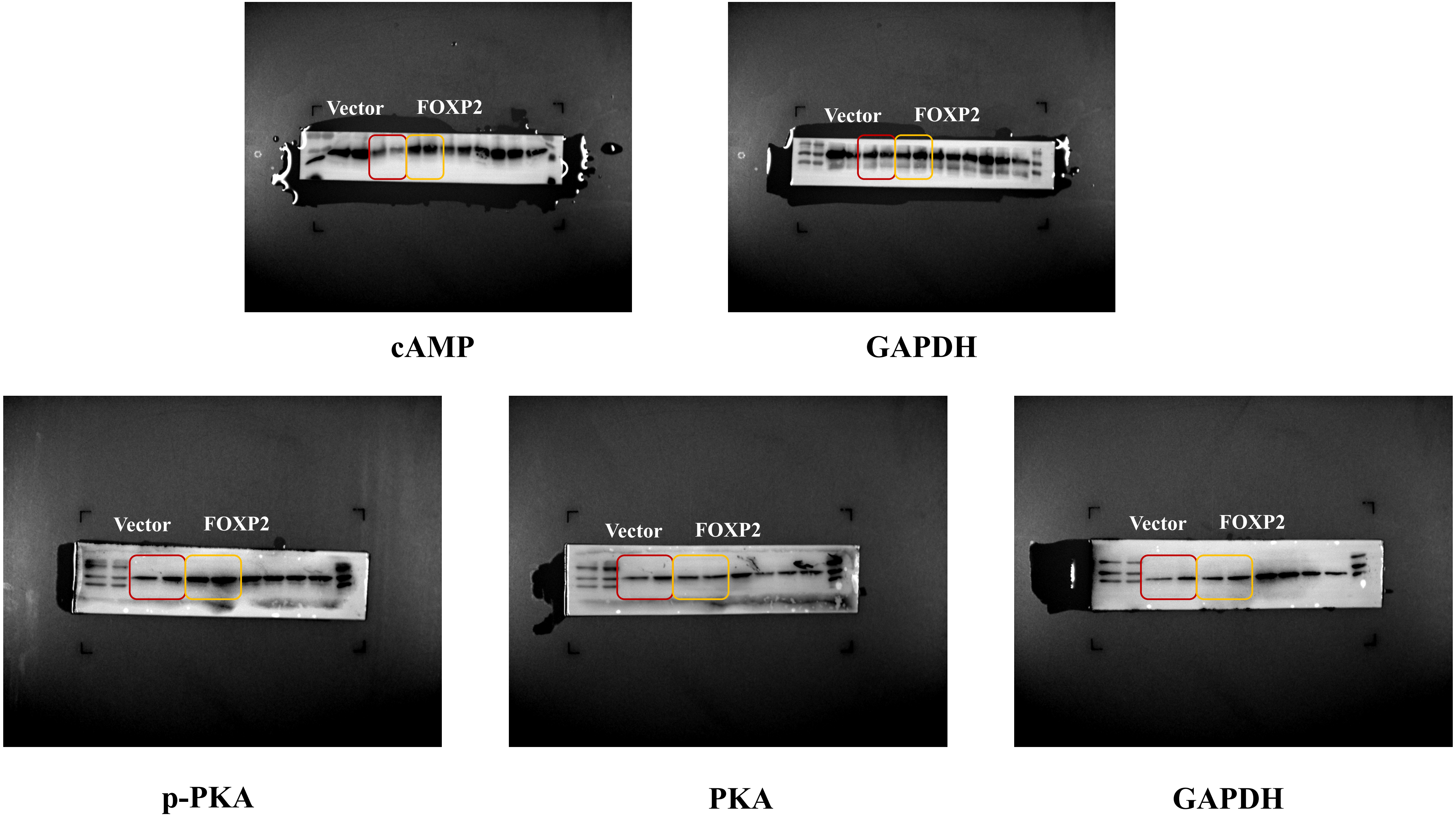

Vector
FOXP2
cAMP
Vector
FOXP2
GAPDH
Vector
FOXP2
p-PKA
Vector
FOXP2
PKA
Vector
FOXP2
GAPDH

Fig.4E, F

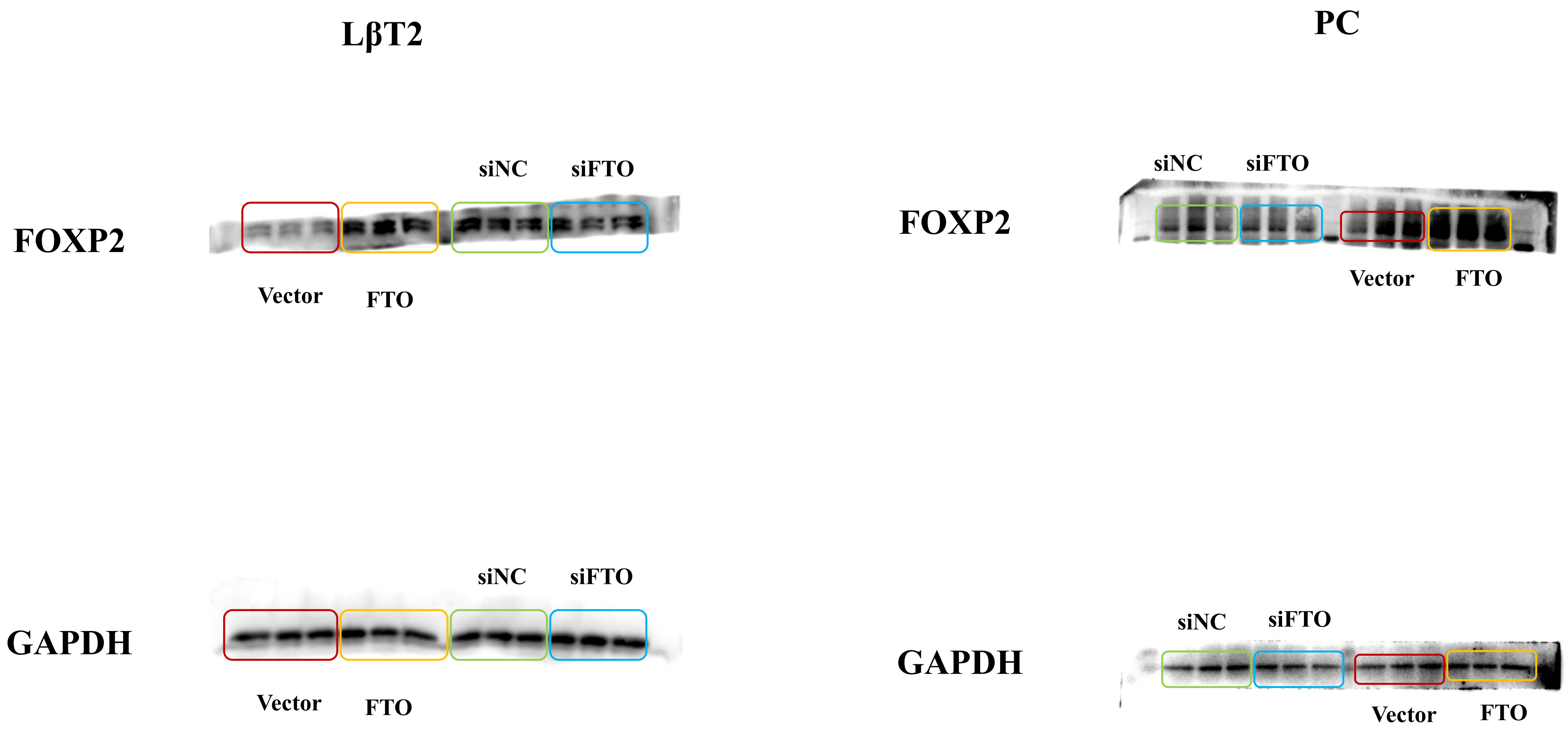
LβT2
PC
FOXP2
siNC
siFTO
Vector
FTO
GAPDH
siNC
siFTO
Vector
FTO
FOXP2
siNC
siFTO
Vector
FTO
GAPDH
siNC
siFTO
Vector
FTO

Fig.S8C

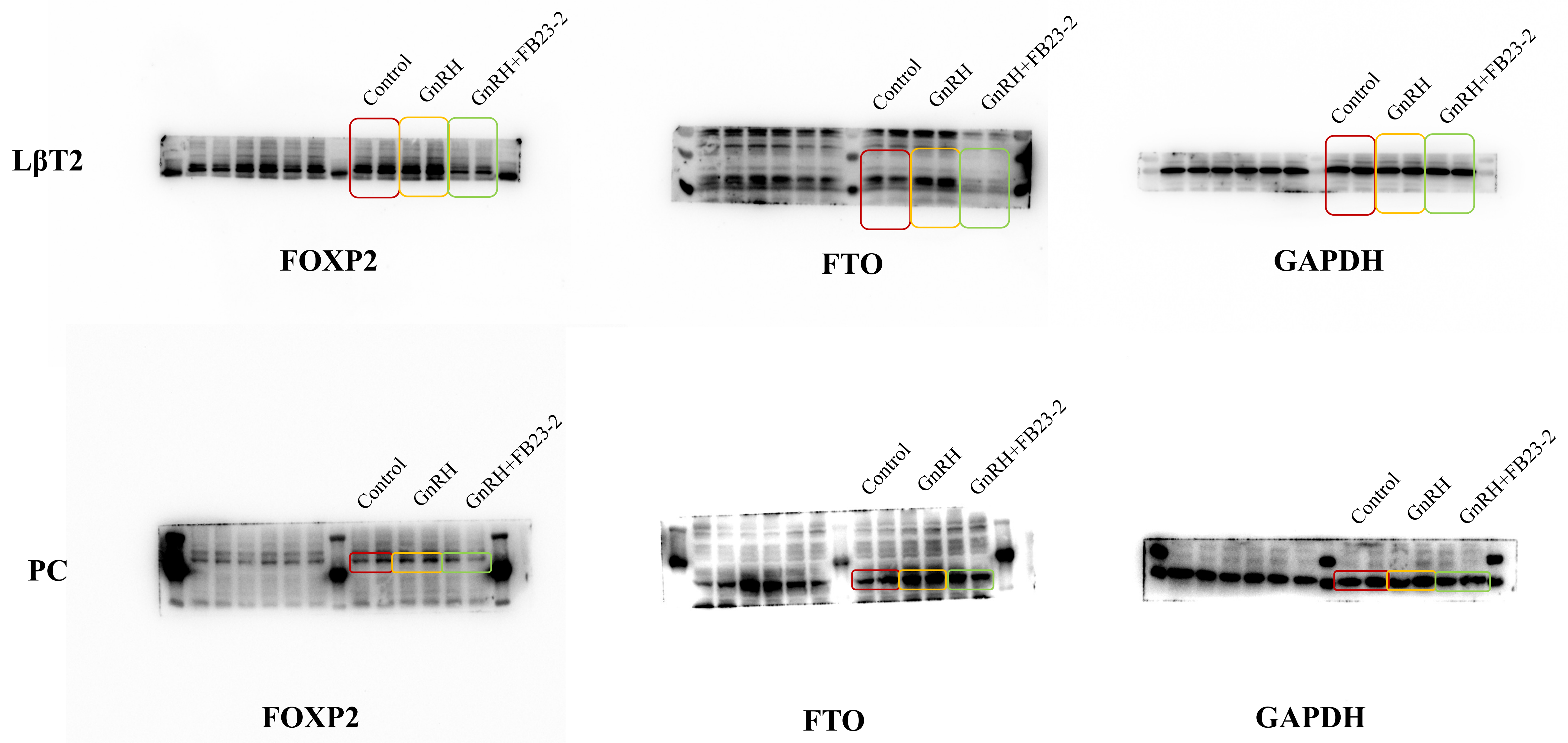

Supplement: Supplementary file 3 — Additional file 3: Raw blot images. [file 12915_2024_1905_MOESM3_ESM.pdf]
